# *Src* Cooperates with Oncogenic *Ras* in Tumourigenesis via the JNK and PI3K Pathways in *Drosophila* epithelial Tissue

**DOI:** 10.3390/ijms19061585

**Published:** 2018-05-27

**Authors:** Carole L.C. Poon, Anthony M. Brumby, Helena E. Richardson

**Affiliations:** 1 Cell Cycle and Development lab, Peter MacCallum Cancer Centre, Melbourne, VIC 3002, Australia; tonybrumby@iinet.net.au; 2 Department of Biochemistry and Molecular Biology, University of Melbourne, Melbourne, VIC 3010, Australia; 3 Department of Anatomy and Cell Biology, University of Melbourne, Melbourne, VIC 3010, Australia; 4 Department of Biochemistry and Genetics, La Trobe Institute of Molecular Science, La Trobe University, Melbourne, VIC 3086, Australia

**Keywords:** Src, Ras, Raf, PTEN, PI3K, cooperative tumourigenesis, *Drosophila*

## Abstract

The *Ras* oncogene (Rat Sarcoma oncogene, a small GTPase) is a key driver of human cancer, however alone it is insufficient to produce malignancy, due to the induction of cell cycle arrest or senescence. In a *Drosophila melanogaster* genetic screen for genes that cooperate with oncogenic *Ras* (bearing the *Ras^V12^* mutation, or *Ras^ACT^*), we identified the *Drosophila Src* (Sarcoma virus oncogene) family non-receptor tyrosine protein kinase genes, *Src42A* and *Src64B*, as promoting increased hyperplasia in a whole epithelial tissue context in the *Drosophila* eye. Moreover, overexpression of *Src* cooperated with *Ras^ACT^* in epithelial cell clones to drive neoplastic tumourigenesis. We found that *Src* overexpression alone activated the Jun N-terminal Kinase (JNK) signalling pathway to promote actin cytoskeletal and cell polarity defects and drive apoptosis, whereas, in cooperation with *Ras^ACT^*, JNK led to a loss of differentiation and an invasive phenotype. *Src + Ras^ACT^* cooperative tumourigenesis was dependent on JNK as well as Phosphoinositide 3-Kinase (PI3K) signalling, suggesting that targeting these pathways might provide novel therapeutic opportunities in cancers dependent on Src and Ras signalling.

## 1. Introduction

The Src (Sarcoma virus oncogene) family of non-receptor tyrosine protein kinases are highly conserved and comprise nine members in vertebrates: Src, Fyn (oncogene related to Src, Fgr, Yes), Yes (*Yamaguchi sarcoma virus* oncogene), Blk (B Lymphoid Tyrosine Kinase), Yrk (Yes-related kinase), Fgr, Hck (hemopoietic cell kinase), Lck (lymphocyte-specific protein tyrosine kinase) and Lyn (v-yes-1 Yamaguchi sarcoma viral related oncogene homolog) [1]. Of these, Src, Fyn and Yes are ubiquitously expressed in tissues and the remaining members are restricted to specific cell types [2]. Src family kinases have pleiotropic functions including intracellular signalling, actin remodelling, cell adhesion and apoptosis [1,3,4,5]. Despite extensive analysis in cell culture and mouse models, the precise role of Src kinases during tumourigenesis in vivo is yet to be clearly defined. Aberrant Src activity is strongly associated with human tumour development [6], and, in analysis of human tumour samples, increased Src activity arises from an activating mutation at the inhibitory C-terminal tyrosine residue [7]. However, other studies indicate that elevated Src activity is due to increased protein expression and increased kinase activity that enhance tyrosine phosphorylation of substrates [6,8,9,10,11,12,13,14,15,16].

Although there is a clear correlation for increased Src function in human cancer, there are discrepancies in the literature regarding the influence of overactivated Src at different stages of tumour development. Aberrant Src activation correlates with advanced cancer development and is associated with tumour characteristics, such as increased invasiveness and metastasis [6,7,15,17]. However, other studies suggest that Src may be required earlier in tumour development. Increased Src activity is observed in samples sourced from low-grade human bladder tumour samples compared with a low Src activity in high grade samples [11]. Interestingly, metastatic cell lines that possess elevated Src kinase activity are more sensitive to receptor tyrosine kinase (RTK) signalling [18], suggesting that Src may require other cooperative events.

Indeed, c-Src (cellular-Src proto-oncogene) cooperates with the epidermal growth factor (EGF) receptor (EGFR) in murine fibroblast cell lines [19], and downstream of EGFR signalling with activated (oncogenic) mutations in the Ras (Rat Sarcoma oncogene) small-GTPase [20,21]. Oncogenic *Ras* mutations (such as *Ras^V12^*) locks Ras in the GTP-bound activated state resulting in constitutive signalling through the MAPK (Mitogen activated protein kinase) pathway [22,23]. The cooperative interaction of Src with EGFR is characterised by increased DNA synthesis and colony formation in soft agar in vitro and increased tumour incidence in vivo when c-Src- and EGFR-expressing cells are transplanted into nude mice [19]. The cooperation is also reflected in three-dimensional cell culture where overexpression of c-Src and EGFR in human epithelial cell lines results in disruption to acinar architecture and mislocalisation of polarity markers resulting in potentiation of invasion, migration and anchorage-independent growth [24]. Additionally, combinatorial treatment with inhibitors of EGFR (Gefitinib) and Src (AZD0530) in human head and neck squamous cell carcinoma cell lines show greater reduction of growth and invasion compared with treatment with a single compound [25]. With the high correlation of EGFR and Src expression in primary human colon cancer cells [18] and mammary breast tumours [13], and oncogenic *Ras, Ras^V12^*, and *Src* in other human cancer cell lines [20,21], these observations suggest that the contributions of both EGFR-Ras and Src are important in cooperative tumourigenesis.

Interestingly, the requirement of Src in tumourigenesis appears to be context dependent. In vitro, c-Src expression alone cannot transform cells without cooperating partners [26,27,28,29], whilst. in an in vivo mouse model, c-Src expression is sufficient to initiate tumour formation [30]. The ubiquitously expressed Src family member, Yes, can activate Ras-MAPK signalling, unlike c-Src in colorectal cancer cells [31], and therefore may require alternate cooperative partners to c-Src. On the other hand, another ubiquitously-expressed Src family kinase, Fyn, is induced by Ras-MAPK signalling and required for the mesenchymal phenotype or invasive behaviour of Ras-driven breast and skin cancer cells [32,33]. These context-dependent functions of Src family members in cancer suggests that analysis of overexpressed or activated Src within a simple in vivo biological context may reveal functions of Src kinases, either alone or with a cooperating partner, that are not readily discerned using in vitro systems or in vivo knockout models. In the vinegar fly, *Drosophila melanogaster*, the two Src family homologues, Src42A and Src64B, are highly conserved in sequence and domain structure with vertebrate c-Src (an overall identity of 61% and 49%, respectively). Thus, in comparison to the nine Src kinases identified in vertebrates [1], *Drosophila* provides an opportunity to study the role of Src function in vivo as there is less complication from functional compensation from multiple Src family members such as observed in mouse knockout models [34,35]. Given the different biological responses when Src is expressed in vitro or in vivo, a whole animal model of tumourigenesis, such as in *Drosophila*, provides an opportunity to investigate the in vivo role of Src kinases and its effectors in the development of cancer.

Previous studies have analysed the role of the two *Drosophila* Src kinase family members, Src42A and Src64B, in different settings in vivo. In the *Drosophila* developing eye, ectopic expression of wild-type *Src64B* results in a disorganised (rough) eye phenotype, due to supernumerary R7 cells [36], although overexpression of wild-type *Src42A* does not have a discernible effect [37]. Furthermore, expression of C-terminally truncated *Src42A* or *Src64B*, rendering constitutive activation, resulted in a more pronounced phenotype [36,37]. Analysis in the *Drosophila* eye revealed that overexpression of Src resulted in different phenotypes dependent on expression level, with strong overexpression resulting in reduced eye size due to increased proliferation accompanied by elevated cell death [38]. However, lower levels of Src activation using a mutation in a negative regulator of Src, C-terminal Src-related kinase (Csk), resulted in tissue overgrowth [39]. *Csk* loss induced overproliferation in the eye epithelium, even within regions of differentiation, suggesting that cells are unable to exit the cell cycle [40,41]. Genetic analysis revealed that the *Csk* mutant overgrowth phenotype was suppressed by mutations in *Src42A* and *Src64B*, as well as the downstream Src kinase, *Btk29A* (*Tec29*) [40]. Furthermore, knockdown of Jun N-terminal Kinase (JNK) and the STAT92E transcription factor suppressed the *Csk* mutant overgrowth phenotype [40]. Furthermore, other studies have revealed that downstream of Src signalling, the impairment of the conserved Hippo negative tissue growth control and tumour suppressor pathway [42,43] is important for Src-induced tissue overgrowth [44,45].

In another context, discrete expression of activated *Src42A* in the embryo results in apoptosis and migration of cells away from the expression domain [46]. A migratory phenotype is also observed with *Csk*-deficient cells in the wing epithelium, where cells are excluded basally from the epithelia, migrating through the extracellular matrix and eventually undergoing apoptosis [47]. This phenotype was only observed at the borders between wild-type and mutant cells, and required input from *E*-cadherin, p120-catenin, RhoA, JNK and matrix metalloproteinases (MMP1/2) [47,48]. This effect is also observed upon Src42A overexpression along the dorsal-ventral boundary in the wing epithelium, which was dependent on JNK activation regulated by the E2 ubiquitin ligase Bendless-dUev1a [49]. The JNK pathway is also a key effector of Src in dorsal closure (the process of epithelial sheet migration to close the dorsal epidermis during embryogenesis), where activation of JNK signalling partially suppresses the dorsal open phenotype associated with *Src42A^−^ Tec29^−^* double mutant flies [50]. Thus, in Src-mediated cell migration/invasion, JNK activation is a key effector of the invasive phenotype [47,48,49,50].

We investigated the role of the *Drosophila* Src kinases in cooperative tumourigenesis with activated (oncogenic) *Ras* (*Ras^V12^* or *Ras^ACT^*) in the fly eye epithelium. Characterisation of *Src42A* and *Src64B* reveals both dose- and context-dependent effects. *Src* expression alone results in increased cell death, a loss of cell polarity and disruption to F-actin organisation, but in itself is not sufficient to promote tumour formation. Significantly, overexpression of *Drosophila Src* genes cooperate with *Ras^ACT^* in eye disc clones, resulting in neoplastic overgrowth characterised by tissue overgrowth, increased clonal tissue size, loss of differentiation, disrupted F-actin organisation and cell polarity, and invasive clonal phenotypes leading to larval lethality. This cooperation requires the contributions of the Raf as well as the Phosphoinositide 3-Kinase (PI3K) effector pathways of Ras. Src activates JNK to promote apoptosis and defects in F-actin, however, when *Ras^ACT^* is coexpressed, JNK pathway signalling contributes to inhibition of differentiation, clonal overgrowth and invasive phenotypes associated with *Src* + *Ras^ACT^* neoplastic overgrowth. Given the strong correlation of aberrant Src function with EGFR-Ras activation in human cancers, the finding that JNK and PI3K are critical mediators of Src–Ras cooperative tumourigenesis may provide specific targets for cancer therapy.

## 2. Results

### 2.1. Src42A Overexpression Enhances the Eyeless-Driven Ras^ACT^ Hyperplastic Eye Phenotype

**Figure 1:**
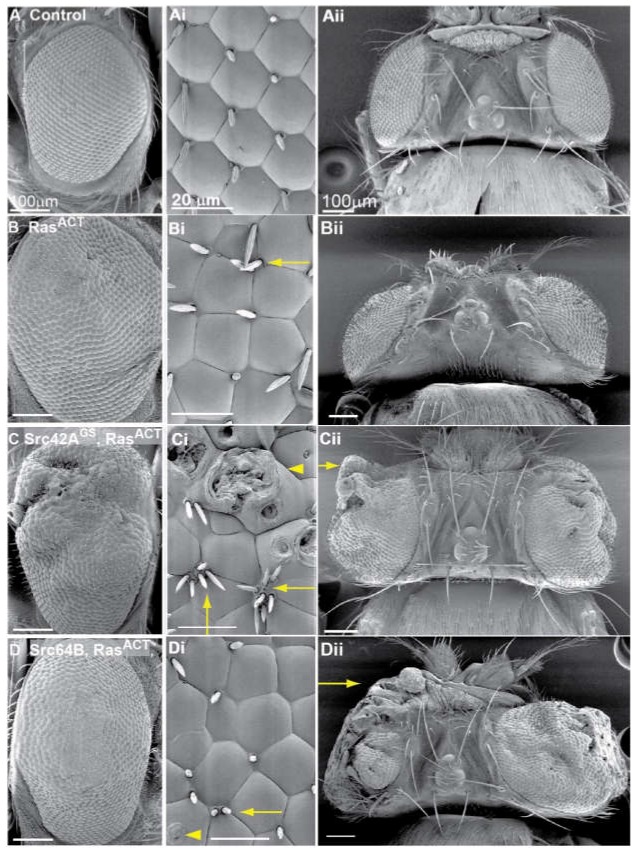
Src enhances hyperplastic *ey > Ras^ACT^* adult eye phenotype. Scanning electron micrographs of male adult flies. Lateral view (first column, 100 μM scale bar) and higher magnification of ommatidia (i, second column, 20 μM scale bar) with posterior to the left. Dorsal view was imaged from a separate example (ii, third column, 100 μM scale bar). Genotypes: (**A**) Control: *ey-GAL4*/+; (**B**) Ras^ACT^: *ey-GAL4*, *UAS-Ras^ACT^*/+; (**C**) Src42A^GS^, Ras^ACT^: *ey-GAL4*, *UAS-Ras^ACT^*/*GS11049;* and (**D**) Src64B, *Ras^ACT^*: *ey-GAL4*, *UAS-Ras^ACT^*/*UAS-Src64B*. Relative to the control (**A**), *ey > Ras^ACT^* (**B**) adult eye were enlarged with irregular ommatidial organisation and ectopic bristles (arrow, **Bi**). Coexpression of *Src42A^GS^* with *Ras^ACT^* (**C**) enhanced the *Ras^ACT^* hyperplastic eye phenotype resulting in enlarged, folded eyes. Adult eyes showed misplaced and ectopic bristles (sometimes in the same vertex, arrows, **Ci**), blistering of ommatidia (arrowhead, **Ci**), and protrusion of the overgrown eye from the head (arrow, **Cii**). *Src64B* expression also enhanced the *ey > Ras^ACT^* hyperplastic phenotype resulting in an enlarged adult eye characterised by enhanced overgrowth and aberrant cuticle (**D**) with ectopic and misplaced bristles (arrow, **Di**), blistering of ommaditia (arrowhead, **Di**), which was less severe in female adults (**D**,**Di**) compared to severe disruption to eye morphology in male adults (arrow, **Dii**).

In a genetic screen designed to identify novel enhancers of *Ras^ACT^*, a *GS* (*Gene Search*) line, *GS11049*, that overexpresses *Src42A* (*Src42A^GS^*) was identified as an enhancer of the hyperplastic *eyeless* (*ey*)*-GAL4*, *UAS (Upsteam Activating Sequence* for GAL4) *-Ras^ACT^* (*ey > Ras^ACT^*) adult eye phenotype [51]. Expression of *Src42A^GS^* with *ey-GAL4* (*ey > Src42A^GS^*, Figure S1B) resulted in a normal eye phenotype relative to the control (Figure 1A and Figure S1A, and Table 1). *ey > Ras^ACT^* expression resulted in a mild hyperplastic rough eye phenotype (Figure 1B and Figure S1K). This phenotype was also characterised by disruption to the ommatidial array, ectopic bristles and misshapen ommaditia (Figure 1Bi). In contrast, expression of *Src42A^GS^* with *ey > Ras^ACT^* enhanced the hyperplastic *Ras^ACT^* eye phenotype resulting in overgrowth of the adult eye (Figure 1C and Figure S1L, and Table 1). Ultrastructural analysis by scanning electron micrographs showed morphological defects in ommatidia organisation, ectopic bristles within one vertex and blistering of ommatidia, most likely due to cone cell defects (Figure 1Ci,Cii). These effects were more severe than observed for *ey > Ras^ACT^* alone (Figure 1B). The dorsal view (Figure 1Cii) showed enhanced outgrowth of *ey > Ras^ACT^ + Src42A^GS^*. The resulting *Src42A^GS^* + *Ras^ACT^* overgrown phenotype was more pronounced in male flies than females.

To validate the interaction identified in the screen, an independent *UAS* line expressing wild-type *Src42A* was tested. *ey-GAL4*-driven expression of *Src42A* alone did not appreciably affect the adult eye (Figure S1C and Table 1), consistent with the *ey > Src42A^GS^* phenotype and previous observations [37]. However, unlike *Src42A^GS^*, coexpression of *Src42A* with *ey > Ras^ACT^* did not enhance the hyperplastic *Ras^ACT^* eye phenotype (Figure S1M and Table 1). Expression of a second wild-type *Src42A* line, *Src42A^EY08937^* (containing a P element insertion, *EPgy2* [52]) with *ey > Ras^ACT^* also did not enhance the hyperplastic *Ras^ACT^* eye phenotype (data not shown). Since previous studies in *Drosophila* have revealed a dosage-dependent response to Src expression [37,38,39], it is possible that positional effects of the *UAS* or *EYgy2* integration site [52,53] may affect expression levels Src, which could explain why they did not enhance the *ey > Ras^ACT^* phenotype. To further analyse the effects of overexpression of *Src42A^GS^* and the independent *UAS-Src42A* line, an alternative driver was adopted. *GMR (Glass Multimer Reporter)-GAL4* drives expression in differentiating cells beginning in the morphogenetic furrow [54,55] in comparison with *ey-GAL4*, which drives expression from stage 15 during embryogenesis and predominates in cycling cells during third instar larval stage [56,57]. *GMR > Src42A^GS^* adult eyes were glazed with loss of pigmentation in the central ommatidia (Figure S2B). In contrast, *GMR > Src42A* adult eyes had a small, glassy stripe in the posterior region of a rough adult eye (Figure S2C). The glassy phenotype was less severe in *GMR > Src42A* than *GMR > Src42A^GS^* adult eyes, suggesting that *Src42A* may not be as potent as *Src42A^GS^* in activation of Src signalling.

To examine Src activity and protein expression levels in the wild-type *Src42A* lines, Western blot analysis was carried out on equally loaded amount of protein lysates from third instar larval heads expressing *hsp70* (*heat-shock inducible promoter)-GAL4* induced *Src42A^GS^* or the *UAS-Src42A* transgene (lanes c and d, respectively, Figure S2D). An antibody raised specifically to *Drosophila* Src42A [58] detected a protein corresponding to endogenous Src42A at an approximate molecular weight of 60 kDa in wild-type lysates (Figure S2D, lane a: no heat shock, lane b: with heat shock). Upon heat shock induction, *Src42A^GS^* lysates and the *UAS-Src42A* transgene lysates showed 2.1-fold and 1.7-fold increase in expression of Src42A, respectively, in comparison to the heat-shocked negative control (Figure S2). The same Western blot was also probed with a phospho-Src (pSrc) antibody, which recognises a conserved phosphorylated Tyrosine residue in the kinase domain and indicates an active Src protein (Figure S2D). Endogenous basal Src activity is evident in negative control lanes (Figure S2D). Upon expression of *Src42A^GS^* or the *UAS-Src42A* transgene, the level of pSrc is increased by 2.5-fold and 1.8-fold, respectively, compared to the negative control (Figure S2D). Taken together, Western blot analysis revealed that expression of *Src42A^GS^* resulted in higher protein levels and autophosphorylated Src compared with *UAS-Src42A*, suggesting that *Src42A^GS^* was more potent than the *UAS-Src42A* transgene in activation of Src signalling. The increased levels of Src expression and activity may explain why *Src42A^GS^*, but not the *UAS-Src42A* transgene, was able to enhance the *Ras^ACT^* hyperplastic eye phenotype.

The C-terminal tail is a crucial regulatory component of all Src family kinases. A conserved Tyrosine residue in this region (Tyr527 in chickens and Tyr530 in humans) when phosphorylated by C-terminal Src-like kinase (Csk) C-terminal Src-like kinase (Csk) and/or Csk-Homologous-Kinase (Chk) mediates Src inhibition [59]. Upon phosphorylation, the kinase folds into a closed, inactive conformation by binding between the C-terminal tail and the SH2 domain, with concomitant binding between the kinase domain and the SH3 domain. This mode of regulation is critical to Src function, most notably illustrated by the v-*Src* (*viral-Src*) mutation, which is rendered constitutively active by truncation of this region [27,60]. Here, malignant potential is enhanced since Src protein is no longer restrained by intramolecular protein interactions. To examine the effect of constitutively active Src, a gain-of-function mutation of Src42 (*Src42A^ACT^*) was utilised, which bears a C-terminal truncation that removes the inhibitory regulatory region resulting in unattenuated Src signalling [37]. Expression of *UAS*-*Src42A^ACT^* alone, using *ey-GAL4*, resulted in a range of adult eye sizes from a reduced eye, a split eye, or a completely absent eye, where cuticle and/or ectopic hairs replaced some areas of the eye field (representative image, Figure S2D and Table 1). The range of phenotypes observed can be attributed to the inherent variability observed with the *ey-GAL4* driver [51]. These effects suggest that *Src42A^ACT^* was more potent in activation of Src signalling than the wild-type *Src42A* transgene tested and, consistent with this idea, expression of *Src42A^ACT^* with the *GMR-GAL4* driver resulted in pupal lethality. However, coexpression of *Src42A^ACT^* + *Ras^ACT^* with *ey-GAL4* did not enhance the hyperplastic *Ras^ACT^* eye phenotype (Figure S1N and Table 1). There was some overgrowth in the dorsal region of the eye but the ventral region appeared reduced (Figure S1N). The phenotypes were more pronounced in males than females, and fewer males eclosed than female adults (1 male in 10 *Src42A^ACT^ + Ras^ACT^* eclosed adults) suggesting a degree of lethality. Altogether, these analyses are consistent with previous observations in *Drosophila* that *Src* expression and activation results in a dosage-dependent response [38,39].

### 2.2. Src64B Overexpression Also Enhances the ey > Ras^ACT^ Hyperplastic Eye Phenotype

A *GS* line overexpressing *Src64B*, *GS9875*, was identified in the genetic screen as a mild enhancer of *ey > Ras^ACT^* [51]. An independent *UAS-Src64B* line was also tested for cooperation with *ey > Ras^ACT^*. Expression of *Src64B* alone with *ey-GAL4* resulted in a range of adult eye sizes, from a smaller eye to no eye (representative image Figure S1E and Table 1). Additionally, severe defects were also present in the head region with excessive cuticle and ectopic antennae. The reduced *ey > Src64B* adult eye (Figure S1E) was comparable to *ey > Src42A^ACT^* (Figure S1D), suggesting that *Src64B* strongly activates Src signalling. Consistent with this, stronger expression of Src64B, using *GMR > Src64B*, resulted in pupal lethality.

Coexpression of *Src64B* with *ey > Ras^ACT^* enhanced the *Ras^ACT^* hyperplastic eye phenotype characterised by disorganised ommatidia, ectopic bristles and a larger eye (Figure 1D and Figure S1O). Dorsal views demonstrated the severe disruption to adult eye structures (Figure 1Dii, arrow, and Table 1) and highlighted the enhanced outgrowth of the *ey > Ras^ACT^ + Src64B* phenotype. These effects were more pronounced in males and resulted in a degree of lethality. This phenotype was comparable to that observed in the *Ras^ACT^* screen with *Src42A^GS^* [51]. Thus, overexpression or activation of Src42A and overexpression of Src64B cooperate with oncogenic Ras in inducing tissue overgrowth (summarised in Table 1).

### 2.3. Ras^ACT^ Signalling Contributes more than just Survival Signals in Cooperation with Src

The Ras pathway is a central hub for cell signalling and in *Drosophila* has been shown to regulate cellular functions including specification, proliferation, growth and cell survival [61,62,63,64,65,66,67]. Although Ras has a multitude of effectors by which it can influence these processes, the Raf-MAPK cascade has predominantly been implicated in many of these functions. For example, Ras-mediated Raf-MAPK signalling results in ectopic proliferation and hyperplastic growth [62], and also promotes cell survival [66,67].

Given the small eye phenotype upon expression of the stronger Src lines (Figure S1D,E) and the previous roles in proliferation and apoptosis ascribed to Src (for example, in [38]), we tested whether the role of Ras^ACT^ in cooperation with Src family kinases was merely to mediate protection from cell death. Firstly, to mimic the function of Ras in promoting cell survival, the cell death inhibitor, the baculovirus protein p35, which acts as a substrate for effector caspases and thereby inhibits apoptosis [68,69], was used. Coexpression of *p35* with *Src42A^ACT^* or *Src64B* via the *ey-GAL4* driver partially rescued the small adult eye phenotype (Figure S1I,J, respectively, and Table 1), although the eye field was still smaller than control (Figure S1A) or *p35* expressed alone (Figure S1E). There was no obvious difference in the adult eye size of *Src42A^GS^* or *Src42A* upon coexpression with *p35* (Figure S1G,H, respectively, and Table 1) compared with *Src42A^GS^* or *Src42A* expressed alone (Figure S1B,C, respectively, and Table 1). The suppression of the *Src42A^ACT^* or *Src64B* small eye phenotype by *p35* expression suggests that *Src* expression promotes apoptosis. However, since only a partial suppression was observed, it suggests that, in addition to anti-apoptotic cues, *Ras^ACT^* is contributing other functions that cooperate with Src expression.

### 2.4. Src and Ras^ACT^ Cooperate to form Overgrown Neoplastic Tumours in the Eye Epithelium

To further investigate the cooperative interaction between Src family kinases and activated Ras during tumour development, we utilized the *ey-FLP (Flippase) MARCM* (Mosaic Analysis with a Repressible Cell Marker) system to generate clones in the developing eye epithelium [70]. *Src* transgenes were coexpressed with *Ras^ACT^* in clones to determine whether neoplastic overgrowth would occur, similar to that previously observed with mutants in the cell polarity genes, *scribbled* (*scrib*), *disc large* (*dlg*) and *lethal-2-giant larvae* (*lgl*) [71,72,73]. Three *Src42A* lines were co-expressed with *Ras^ACT^* in eye disc clones: the original candidate identified, *Src42A^GS^*; a second wild-type allele, *UAS-Src42A*; and the activated form, *UAS-Src42A^ACT^*. The second Src family member *Src64B* was also tested with *Ras^ACT^* in clones. To establish whether expression of *Src* with *Ras^ACT^* in clones resulted in neoplastic overgrowth, mosaic eye discs were analysed for changes in clonal tissue size, differentiation and F-actin organisation.

**Figure 2:**
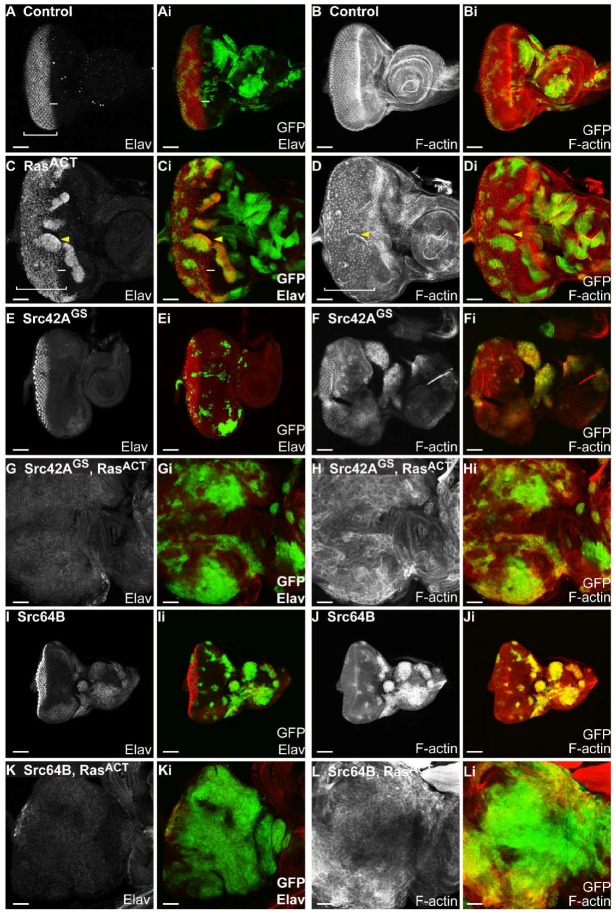
*Src* and *Ras^ACT^* cooperate in clonal analysis of eye disc clones. Confocal images, planar views, of third instar eye-antennal imaginal discs with posterior to the left, in this and subsequent eye antennal imaginal disc figures. GFP (Green Fluorescent Protein) marked clones (green in merged images) were generated using *ey-FLP MARCM* in this and subsequent figures. Elav marks differentiated cells (red in merged images) and rhodamine-phalloidin visualises F-actin to mark cell outlines (red in merged images). Scale bar, 50 μM. The small bars in A,C indicate the morphogenetic furrow, and the brackets in **A**,**C**,**D** indicate the differentiated region of the eye disc. Genotypes: (**A**,**B**) Control: *ey-FLP1*, *UAS-mCD8-GFP/+*; +/+; *tub-GAL4 FRT82B tub-GAL80/FRT82B*; (**C**,**D**) Ras^ACT^: *ey-FLP1*, *UAS-mCD8-GFP/+*; *UAS-Ras^ACT^*/+; *tub-GAL4 FRT82B tub-GAL80/FRT82B*; (**E**,**F**) Src42A^GS^: *ey-FLP1*, *UAS-mCD8-GFP/+*; *GS11049/+*; *tub-GAL4 FRT82B tub-GAL80/FRT82B*; (**G**,**H**) Src42A^GS^, Ras^ACT^: *ey-FLP1*, *UAS-mCD8-GFP/+*; *GS11049*, *UAS-Ras^ACT^*/+; *tub-GAL4 FRT82B tub-GAL80/FRT82B*; (**I**,**J**) Src64B: *ey-FLP1*, *UAS-mCD8-GFP/+*; *UAS-Src64B/+*; *tub-GAL4 FRT82B tub-GAL80/FRT82B*; and (**K**,**L**) Src64B, Ras^ACT^: *ey-FLP1*, *UAS-mCD8-GFP/+*; *UAS-Src64B*, *UAS-Ras^ACT^*/+; *tub-GAL4 FRT82B tub-GAL80/FRT82B*. In comparison to control eye discs (**A**,**B**), expression of *Ras^ACT^* resulted in clones with rounded borders (arrowhead, GFP, **C**) and ectopic differentiation within clonal tissue (arrowhead, **C**) located just anterior to the morphogenetic furrow (small bar, **C**). Tissue morphology was disrupted, resulting in an enrichment of F-actin in the wild-type tissue surrounding rounded clones (yellow arrowheads, **D**), and a disruption in the regular array of photoreceptors and ommatidial clusters (bracket, **D**). Compared to clones in control (**A**,**B**), *Ras^ACT^* (**C**,**D**), *Src42A^GS^* (**E**,**F**) or *Src64B* (**I**,**J**) mosaic eye discs, expression of *Src42A^GS^ + Ras^ACT^* (**G**,**H**) or *Src64B + Ras^ACT^* (**K**,**L**) in mosaic eye discs resulted in clone and tissue overgrowth. *Src42A^GS^ + Ras^ACT^* clonal tissue was overgrown (**G**,**H**), but did not overtake the eye disc. In contrast, *Src64B + Ras^ACT^* clonal tissue consistently comprised most of the eye imaginal disc tissue (**K**,**L**) in comparison to *Ras^ACT^* (**C**,**D**) or *Src64B* clones (**I**,**J**). Coexpression of *Src42A^GS^ + Ras^ACT^* (**G**,**H**) or *Src64B + Ras^ACT^* (**K**,**L**) resulted in a loss of differentiation (**G**,**K**, respectively), and disruption of F-actin organisation and accumulation of F-actin in clonal tissue (**H**,**L**, respectively). Cell morphology was altered and normal photoreceptors (as outlined by F-actin) could not be distinguished (**H**,**L**).

Expression of *Ras^ACT^* alone in mosaic eye discs resulted in clones with rounded borders (Figure 2C,D) similar to that observed previously [64] compared to the jagged edges of clones in control discs (Figure 2A,B). Compared with the regular array of photoreceptors in the posterior region in the control mosaic eye disc (Figure 2A), expression of *Ras^ACT^* resulted in ectopic differentiation within clonal tissue (Figure 2C) located just anterior to the morphogenetic furrow (bar, Figure 2C). Spacing between ommatidial clusters was irregular in *Ras^ACT^*-expressing mosaic eye discs (Figure 2C,D). The tissue was folded resulting in an apparent enrichment of F-actin at the borders between wild-type and clonal tissue (Figure 2D). These observations correlate with previous clonal analysis of *Ras^ACT^* in the eye disc that showed ectopic differentiation and rounded borders [51,64,74]. The expression of *Ras^ACT^* in eye disc clones resulted in pupal lethality.

Expression of *Src42A^GS^* (Figure 2E,F) or *Src42A^ACT^* (Figure S3D) resulted in dramatically reduced Src-expressing clonal size compared to control mosaic eye discs (Figure 2A,B). In contrast, expression of the weaker wild-type *Src42A* transgene (Figure S3A) resulted in similarly sized clonal tissue to the control (Figure 2A,B). Despite the differences in clonal tissue size, expression of the three *Src42A* transgenes resulted in similar mosaic adult eyes to each other and to the control (Figure S5L,N), indicating the possibility that mutant tissue was eliminated by the adult stage. *Src64B* mosaic eye discs contained large clonal clusters that predominantly localised to the anterior of the eye disc or the antennal disc (Figure 2I). The expression of *Src42A^GS^* or *Src64B* in eye disc clones also delayed development resulting in adults that eclosed 1–2 days after their control counterparts. In *Src64B* mosaic animals, black masses were observed throughout the body of larvae and adults. These black masses are likely to be melanotic tumours arising from an immune response. Melanotic tumours have previously been observed in *Drosophila* larvae in the context of loss of a caspase protein, *Drosophila* Caspase-1 (*dcp*) [75], as well as haemopoietic defects upon overproliferation, arising from activation of the JAK/STAT pathway [76,77].

Coexpression of *Src42A^GS^* + *Ras^ACT^* in clones (Figure 2G,H and Table 2) resulted in tissue overgrowth, where the eye antennal disc was greatly enlarged compared with wild-type (Figure 2A,B), *Ras^ACT^* (Figure 2C,D) or *Src42A^GS^* (Figure 2E,F) control eye discs. Compared to the flat, planar shape of control mosaic eye discs, *Src42A^GS^* + *Ras^ACT^* mosaic eye discs formed a three-dimensional amorphous mass of tissue (Figure 2G,H). During late third instar stages, the eye and antennal structures were no longer distinguishable (Figure 2G,H). These tissues fused together and, in rare instances, the eye-antennal imaginal disc also fused with the brain lobe. Although *Src42A^GS^* + *Ras^ACT^* mosaic eye discs were larger than the controls, *Src42A^GS^ + Ras^ACT^* clonal tissue did not predominate in the eye disc, suggesting that non-cell autonomous overgrowth was also occurring (Figure 2G,H). There was also a loss of differentiation, marked by Elav, within the eye disc in both clonal and surrounding wild-type tissue (Figure 2G). *Src42A^GS^ + Ras^ACT^* clonal tissue showed higher accumulation of F-actin and an overall disruption to F-actin organisation within the eye tissue was observed (Figure 2H). Furthermore, *Src42A^GS^ + Ras^ACT^* larvae were larger in size than controls and reached third instar later at Day 6–7, rather than at Day 5 as observed for controls, with melanotic masses in the abdomen and subsequent lethality during late third instar. Taken together, the overgrowth, loss of differentiation, and altered cell morphology of clonal tissue suggests that expression of *Src42A^GS^ + Ras^ACT^* in eye disc clones induces neoplastic tumour formation. Similar effects were also observed with expression of *UAS-Src42A* with *Ras^ACT^* (Figure S3B,C), however differentiation was still observed in the apical section of wild-type tissue although the patterning was disrupted (Figure S3B), and differentiating clonal and wild-type tissue was also observed aberrantly in basal sections (Figure S3C).

**Figure 3:**
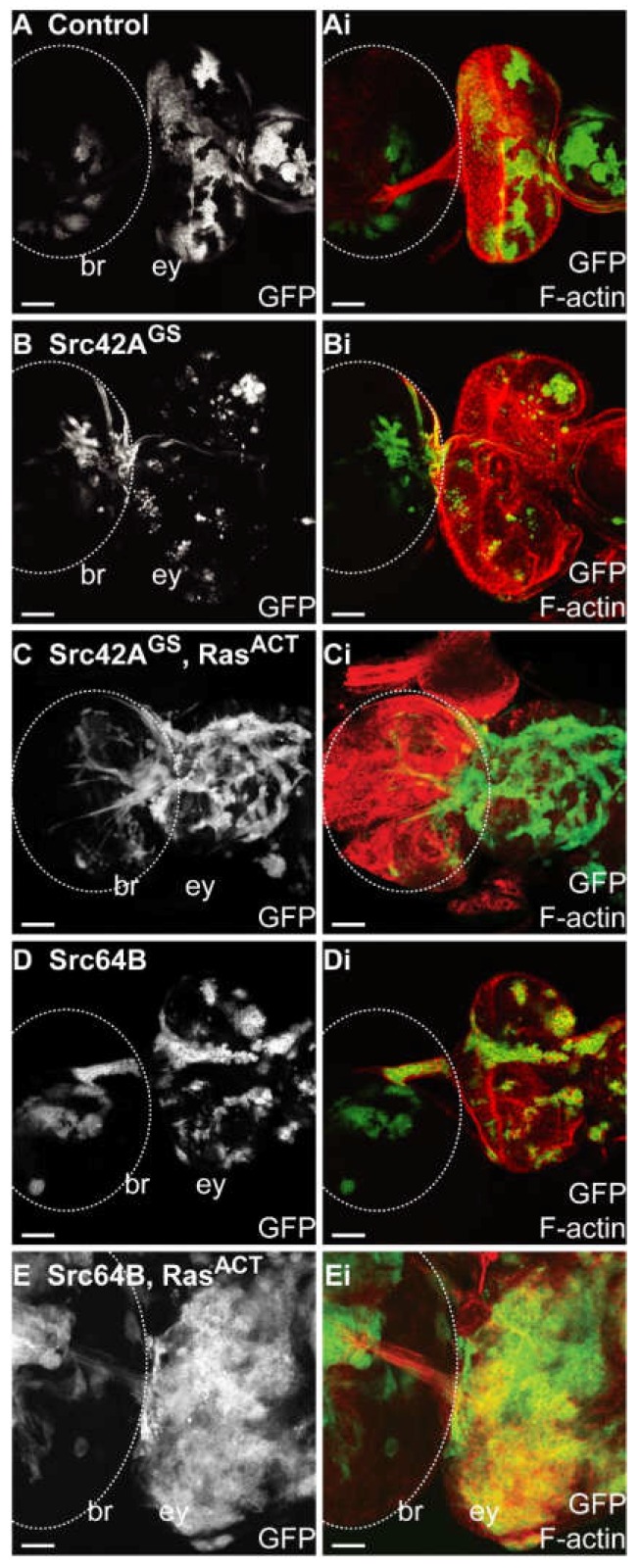
Expression of *Src42A^GS^ + Ras^ACT^* results in protrusive morphology of eye disc clones. Confocal images of eye discs attached to the brain lobes. Clones are marked by GFP (green in merged images). Rhodamine-phalloidin visualises F-actin (red in merged images). Scale bar, 100 μM. The white dashed circle indicates the brain lobe region. Genotypes: (**A**) Control: *ey-FLP1*, *UAS-mCD8-GFP/+*; +/+; *tub-GAL4 FRT82B tub-GAL80/FRT82B*; (**B**) Src42A^GS^: *ey-FLP1*, *UAS-mCD8-GFP/+*; *GS11049/+*; *tub-GAL4 FRT82B tub-GAL80/FRT82B*; (**C**) Src42A^GS^, Ras^ACT^: *ey-FLP1*, *UAS-mCD8-GFP/+*; *GS11049*, *UAS-Ras^ACT^*/+; *tub-GAL4 FRT82B tub-GAL80/FRT82B*; (**D**) Src64B: *ey-FLP1*, *UAS-mCD8-GFP/+*; *UAS-Src64B/+*; *tub-GAL4 FRT82B tub-GAL80/FRT82B*; and (**E**) Src64B, Ras^ACT^: *ey-FLP1*, *UAS-mCD8-GFP/+*; *UAS-Src64B*, *UAS-Ras^ACT^*/+; *tub-GAL4 FRT82B tub-GAL80/FRT82B*. In comparison to control eye discs and brain lobes (**A**), expression of *Src42A^GS^* resulted in reduced clonal tissue in the eye disc and clonal tissue with protrusive morphology in the brain lobe (**B**). *Src42A^GS^ + Ras^ACT^*-expressing clones in the brain lobe (br, **C**) were observed to have an enhanced protrusive morphology compared to brain lobes adjacent to mosaic eye discs expressing *Src42A^GS^* alone (br, **B**). Expression of *Src64B* resulted in small clones in the eye disc (ey, **D**) and adjacent brain lobe (br, **D**). Expression of *Src64B + Ras^ACT^* resulted in large clones in the eye disc and in the brain lobe (br, **E**).

Expression of *Src42A^ACT^ + Ras^ACT^* resulted in overgrowth of the eye antennal disc (Figure S3E) with severe disruption to its planar structure and tissue morphology in comparison to wild-type (Figure 2A,B), *Src42A^ACT^* (Figure S3D) or *Ras^ACT^* (Figure 2C,D) controls. In contrast to either of the *Src42A* lines expressed with *Ras^ACT^*, the activated *Src42A^ACT^ + Ras^ACT^* GFP-marked clonal tissue comprised the majority of the eye imaginal disc (Figure S3E). Clones formed in rounded clusters (Figure S3E) and large clones predominantly localised to the basal part of the epithelium. Differentiation was observed in small regions of wild-type tissue, but within *Src42A^ACT^ + Ras^ACT^* clonal tissue, differentiation was greatly reduced (Figure S3Ei). In *Src42A^ACT^ + Ras^ACT^* clones, F-actin levels were enriched and F-actin organisation was severely disrupted compared to adjacent wild-type tissue (Figure S3Eii,Eiii). The normal ommatidial clusters in the posterior region could no longer be distinguished. As observed for expression of *Src42A^GS^ + Ras^ACT^* (Figure 2G,H) or *Src42A + Ras^ACT^* (Figure S3B,C), expression of *Src42A^ACT^ + Ras^ACT^* resulted in delayed development and lethality at late third instar. In addition to the tissue overgrowth observed in the eye disc, GFP-positive tissue was observed in the brain lobes of *Src42A^GS^* (Figure 3C), *Src42A* or *Src42A^ACT^* transgenes (Figure S3F,G) coexpressed with *Ras^ACT^*. In the brain lobe, clonal tissue expressing *Src42A^GS^* alone also had a protrusive clonal morphology (Figure 3B), but this phenotype was enhanced upon further expression of *Ras^ACT^* (Figure 3C). 

Coexpression of *Src64B + Ras^ACT^* in eye disc clones also resulted in enhanced overgrowth of the eye-antennal imaginal disc (Figure 2K,L and Table 3). *Src64B + Ras^ACT^* clonal tissue encompassed most of the eye imaginal disc and clonal mutant size was increased compared to wild-type control (Figure 2A,B), *Ras^ACT^* (Figure 2C,D) or *Src64B* mosaic eye discs (Figure 2I,J). Differentiation was absent in both clonal and wild-type tissue, although there were rare examples of differentiated cells in wild-type tissue (Figure 2K,L). F-actin levels were enriched within clones and F-actin organisation was disrupted in the eye tissue (Figure 2L). *Src64B + Ras^ACT^* larvae developed more slowly than control counterparts, reaching third instar at Day 6 or 7, compared with Day 5 for controls, and subsequently died during late third instar. Unlike that observed for *Src42A* transgenes + *Ras^ACT^*, expression of *Src64B* alone (Figure 3D) or *Src64B + Ras^ACT^* did not result in clones with protrusive morphology in the brain lobe (Figure 3E).

In summary, in the clonal system, expression of Src42A or Src64B cooperated with *Ras^ACT^* to result in neoplastic overgrowth (summarised in Table 2 and Table 3). *Src + Ras^ACT^* mosaic eye discs were characterised by overgrowth of the eye antennal tissue, an increase in clonal tissue, loss of differentiation and disruption to F-actin organisation. The cooperation observed between *Drosophila Src* genes and *Ras^ACT^* in the clonal system therefore validates the *ey > Ras^ACT^* screen [51] for identifying cooperating partners in tumourigenesis.

### 2.5. Expression of Drosophila Src42A and Src64B Results in Distinct Effects in Eye Epithelial Clones

**Figure 4:**
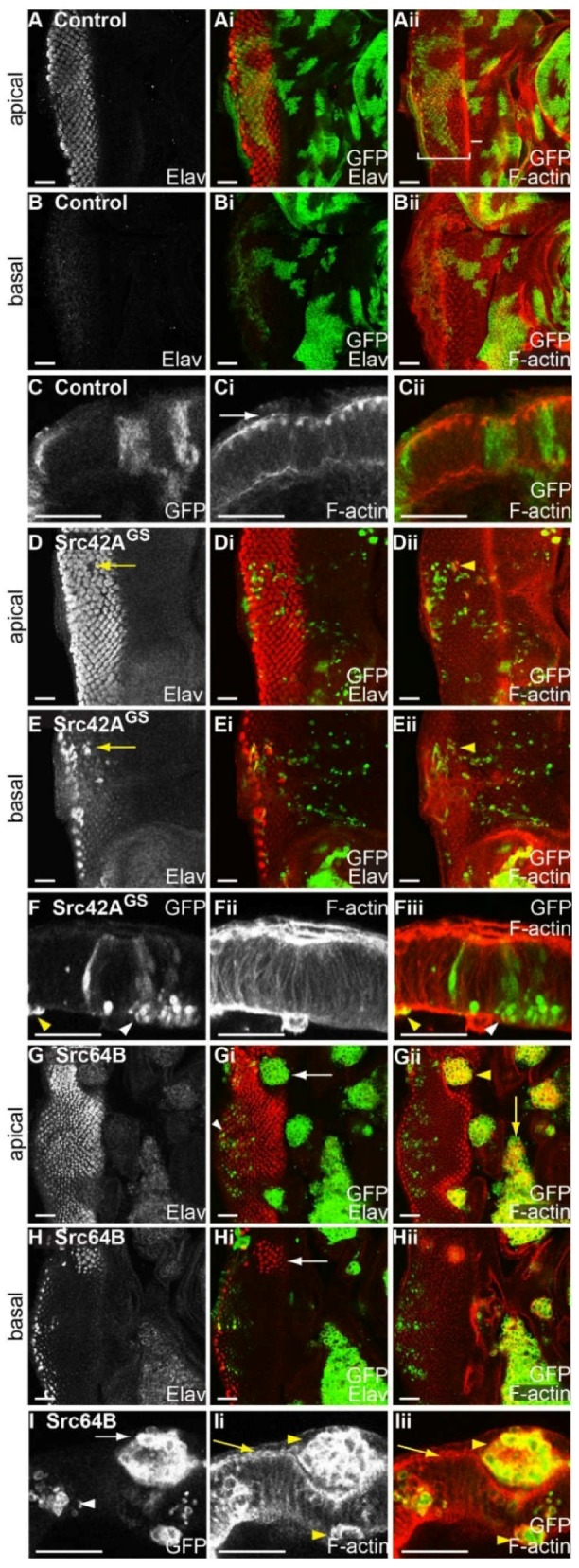
Expression of *Drosophila Src* genes results in distinct effects in eye disc clones. Confocal images, planar views or cross sections, of eye-antennal discs: (**C**,**F**,**I**) posterior to the left. Clones are marked by GFP (green in merged images). Elav marks differentiated cells (in **A**,**B**,**D**,**E,G**,**H** as marked, red in merged images, **ii**) and rhodamine-phalloidin visualises F-actin to mark cell outlines (in **A**,**B**,**D**,**E**,**G**,**H** as marked, red in merged images, **ii**). Apical and basal sections in planar views as marked. Cross sections (**C**,**F**,**I**) represent side mounted eye discs and were oriented with apical to the top, basal to the bottom, and posterior to the left. Cross sections were stained with rhodamine-phalloidin to visualise F-actin (red in merged images). Scale bar, 25 μM. Genotypes: (**A**–**C**) Control: *ey-FLP1*, *UAS-mCD8-GFP/+*; +/+; *tub-GAL4 FRT82B tub-GAL80/FRT82B*; (**D**–**F**) Src42A^GS^: *ey-FLP1*, *UAS-mCD8-GFP/+*; *GS11049/+*; *tub-GAL4 FRT82B tub-GAL80/FRT82B*; and (**G**–**I**) Src64B: *ey-FLP1*, *UAS-mCD8-GFP/+*; *UAS-Src64B/+*; *tub-GAL4 FRT82B tub-GAL80/FRT82B*. Compared to control mosaic eye discs (**A**), expression of *Src42A^GS^* in eye disc clones (**D**–**F**) resulted in greatly reduced clone size (GFP in merged images, **D**,**Ei**,**Eii**). Control eye discs show differentiated cells (Elav) at the apical region of the epithelial cell (compare apical section, **Ai**, to basal section, **Bi**). However, in *Src42A^GS^* mosaic eye discs, small gaps in the differentiation pattern in the apical section of the eye disc were observed (arrow, **D**), which corresponded to clonal tissue where the normally apical Elav staining was now basally located (arrow, **E**). F-actin was enriched in the apical section immediately surrounding these gaps (yellow arrowhead, **Dii**) but not in the basal section (yellow arrowhead, **Eii**). *Src42A^GS^*-expressing clones contained rounded cells that localised to the basal part of the epithelium, sometimes in clusters (white arrowhead, **F**). F-actin was enriched at the apical surface of the epithelium and was generally unperturbed in basal clonal tissue, as observed for controls (**A**), although rare cells within *Src42A^GS^* clones were enriched for F-actin (yellow arrowheads, **F**,**Fiii**). Expression of *Src64B* in eye disc clones (**G**–**I**) resulted in rounded clones located around the morphogenetic furrow and in the anterior (white arrow, **Gi**), which localised discretely above the differentiating epithelium (white arrow, **I**). Note the large apically located cluster (white arrow, **I**) and smaller clusters within the columnar epithelium itself (white arrowhead, **I**). Larger clones were observed in between the eye and antennal imaginal disc (yellow arrow, **Gii**). In wild-type tissue of *Src64B* mosaic eye discs, F-actin was concentrated at the apical surface (yellow arrow, **Ii,Iii**), however, in *Src64B*-expressing clonal tissue, F-actin was increased in clonal tissue and outlined the rounded cells within clonal clusters (yellow arrowheads, **Ii**) in comparison to adjacent wild-type tissue that showed apically enriched F-actin (yellow arrow, **Ii**).

Since all *Src* transgenes tested were confirmed to cooperate with Ras^ACT^ in the clonal system, the expression of Src alone in eye disc clones was characterised for effects on differentiation and F-actin organisation. Compared with control mosaic eye discs (Figure 4A–C), *Src42A^GS^* clones were greatly reduced in size (Figure 4D–F). Differentiation occurred normally in both wild-type and clonal tissue in the mosaic eye disc, however small gaps were observed in apical sections of *Src42A^GS^*-expressing clonal tissue (Figure 4D, yellow arrow). Basal sections of the same eye disc indicated the normally apical photoreceptor nuclei were inappropriately located at the base of the epithelium suggesting that cells within *Src42A^GS^*-expressing clones may be shorter than the adjacent wild-type tissue (Figure 4E, arrow). An accumulation of punctate F-actin was observed apically, but not basally, immediately surrounding these gaps in differentiation (yellow arrowhead, Figure 4Dii). This suggested that the adjacent wild-type tissue could be folding in towards the shorter *Src42A^GS^* clone. Cross sections of *Src42A^GS^*-expressing mosaic eye discs indicated that the small round clones localised basally, sometimes in clusters (Figure 4F), rather than spanning the apical/basal axis of the epithelium as in control discs (Figure 4C). Some *Src42A^GS^*-expressing mosaic eye discs contained clonal tissue clustered beneath the differentiating epithelium. Other than in association with the differentiation gaps, F-actin was generally unaffected in *Src42A^GS^*-expressing eye disc clones, however, rare small round cells were enriched for F-actin (Figure 4F,Fiii, yellow arrowhead).

Expression of *Src64B* in mosaic eye discs resulted in clonal tissue with rounded borders that were excluded from the epithelium (Figure 4G,H). Rounded clones were located around the morphogenetic furrow and in the anterior (Figure 4G, arrow), and larger clones were observed between the eye and the antennal disc (Figure 4Gii, yellow arrow). The apically-localised, rounded *Src64B*-expressing clones did not differentiate (Figure 4Gi, arrow). However, underneath the rounded clones, differentiation occurred normally in the wild-type tissue, as well as in small clones in the epithelium proper (Figure 4Gi, arrowhead). To examine cell shape, *Src64B* mosaic eye discs were stained for F-actin. Planar views (Figure 4Gii,Hii) and cross sections (Figure 4I) of *Src64B*-expressing clones showed an increase in F-actin (Figure 4Gii,Ii,Iii, yellow arrowheads). Wild-type tissue showed apically enriched F-actin (Figure 4I, yellow arrow) as observed in controls (Figure 4Ci arrow). However, the rounded cells within *Src64B*-expressing clones were outlined by increased F-actin (Figure 4Gii, yellow arrowhead, Figure 4Ii,Iii, yellow arrowheads). Generally, *Src64B*-expressing clones were located above the differentiating epithelium (Figure 4I, arrow) and did not span the apical/basal axis of the epithelium (Figure 4I), as observed in control mosaic eye discs (Figure 4C). However, smaller clones were also observed within the epithelium proper (Figure 4I, white arrowhead). Thus, a comparison between *Src42A* and *Src64B* expression in eye disc clones has revealed distinct phenotypic consequences.

### 2.6. Expression of Src64B in Eye Disc Clones Results in A Loss of Cell Polarity

The rounded morphology of cells in *Src42A^GS^* and *Src64B* clonal tissue/clusters suggests a loss of cell polarity and, indeed, Src has an established role in regulation of cell adhesion components [46]. To further analyse the localisation of cell polarity components at a cellular level in *Src*-expressing clones, *Src64B*-expressing mosaic eye discs were stained with the adherens junctions marker *E*-cadherin [78], Discs large (Dlg) which marks septate junctions [78] and subapical proteins, Bazooka (Baz [79]) and atypical protein kinase C (aPKC [79]) (Figure S4). In control mosaic eye discs, *E*-cadherin was localised near the apical surface (Figure S4A). Expression of *Src64B* resulted in diffuse *E*-cadherin localisation within clonal tissue (Figure S4B, white arrow). Larger clones showed *E*-cadherin at the cell surface, but this was not always uniform (Figure S4Bii, arrow, and S4Cii) and while most small, rounded clones within the epithelia did not show aberrant *E*-cadherin localisation, some smaller clones within the epithelia were aberrantly outlined with *E*-cadherin (Figure S4Bii, arrowhead).

In cross-sections of control mosaic eye discs, aPKC (Figure S4D) and Baz (Figure S4Fii) localised to the subapical region, and Dlg (Figure S4Fi) to the septate junction. In *Src64B*-expressing clones within the epithelium proper, aPKC (Figure S4Ei, arrow), Baz (Figure S4Gii, arrow) and Dlg (Figure S4Gi, arrow) were correctly localised in smaller clones that were generally restricted to the posterior region of the eye disc. However, in the large round *Src64B*-expressing clones that were excluded from the epithelium, the subapical markers, aPKC and Baz, were mislocalised (Figure S4Ei, arrowhead and Figure S4Gii). The septate junction protein, Dlg, appeared diffuse in *Src64B*-expressing clonal tissue, although in some cells, a distinct enrichment of Dlg was observed in the cellular cortex (Figure S4Gi, arrowhead). Thus, *Src64B*-expressing large, rounded clonal tissue showed mislocalisation of components of the adherens junctions, septate junctions and subapical complex, which are normally associated with the plasma membrane but become diffuse within the cells of the mutant clone. In contrast, in smaller *Src64B*-expressing clones that span the apical/basal axis of the epithelium, the localisation of cell adhesion components was unaffected relative to the adjacent wild-type tissue.

### 2.7. Expression of Src in Eye Epithelial Clones Promotes Cell Death, but Does Not Reduce Cell Proliferation

In the *Drosophila* eye epithelium, *Src* expression promotes both proliferative and pro-apoptosis signals [38]. To determine the effect of *Src* expression on S phases, BrdU incorporation assays were performed on mosaic eye discs. Expression of *Src42A^GS^* (Figure S5G) or *Src64B* (Figure S5I) did not discernibly affect the pattern of S phases in either mutant clones or surrounding wild-type tissue in the vicinity of the second mitotic wave or in the posterior of the eye epithelium, where differentiation occurs. In rare instances, larger *Src42A^GS^*-expressing clones showed increased S phases anterior to the morphogenetic furrow.

**Figure 5:**
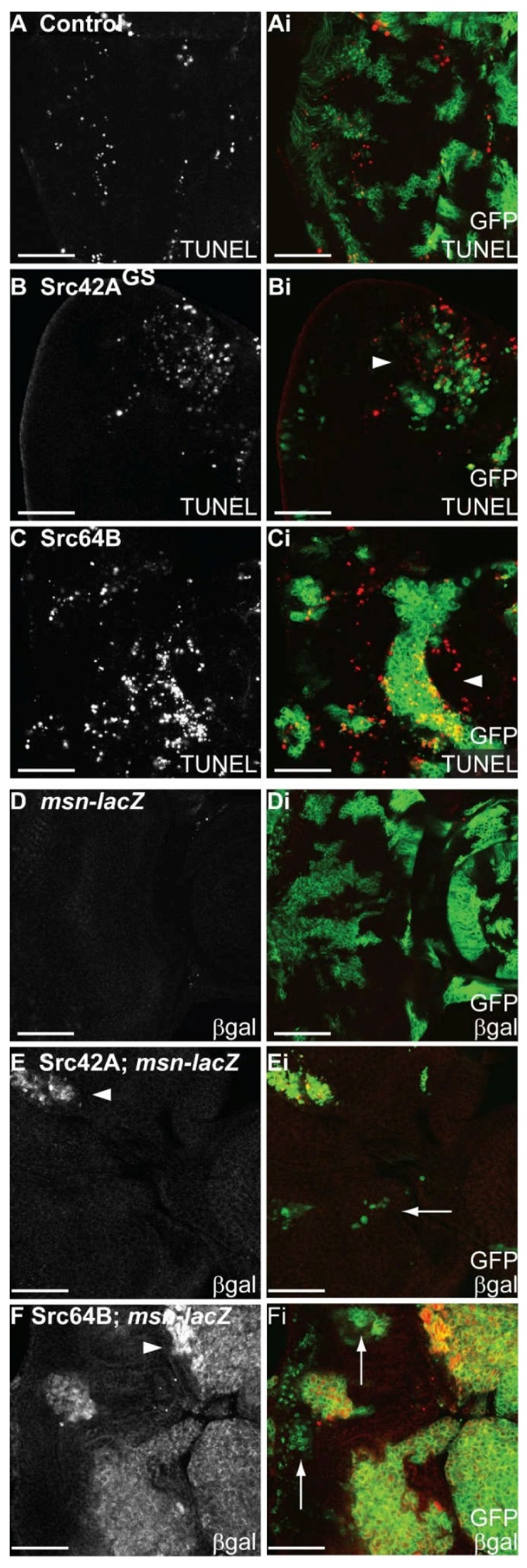
Overexpression of *Src42A^GS^* or *Src64B* results in increased cell death in eye disc clones and JNK pathway activation. Planar confocal images of eye discs: (**A**–**C**) TUNEL assay to analyse cell death (red in merged images); and (**D**–**F**) βgal antibody detection measures transcription of the *msn-lacZ* enhancer trap (red in merged images). Clones are marked by GFP (green in merged images). Scale bar, 50 μM. Genotypes: (**A**) Control: *ey-FLP1*, *UAS-mCD8-GFP*; +/+; *tub-GAL4 FRT82B tub-GAL80/FRT82B*; (**B**) Src42A^GS^: *ey-FLP1*, *UAS-mCD8-GFP/+*; *GS11049/+*; *tub-GAL4 FRT82B tub-GAL80/FRT82B*; (**C**) Src64B: *ey-FLP1*, *UAS-mCD8-GFP/+*; *UAS-Src64B/+*; *tub-GAL4 FRT82B tub-GAL80/FRT82B*; (**D**) *msn-lacZ* control: *ey-FLP1*, *UAS-mCD8-GFP/+; +/+; tub-GAL4 FRT82B tub-GAL80/msn-lacZ FRT82B*; (**E**) Src42A^GS^; *msn-lacZ*: *ey-FLP1*, *UAS-mCD8-GFP/+; GS11049/+; tub-GAL4 FRT82B tub-GAL80/msn-lacZ FRT82B*; and (**F**) Src64B; *msn-lacZ*: *ey-FLP1*, *UAS-mCD8-GFP/+; UAS-Src64B/+; tub-GAL4 FRT82B tub-GAL80/msn-lacZ FRT82B*. In comparison to control mosaic discs (**A**), expression of *Src42A^GS^* (**B**) or *Src64B* (**C**) resulted in an increased number of TUNEL positive cells. The apoptotic cells appeared around and within *Src42A^GS^* (arrowhead, **Bi**) and *Src64B*-expressing clones (arrowhead, **Ci**). JNK pathway activation, as measured by the *msn-lacZ* enhancer trap (**D**–**F**), in control mosaic eye imaginal discs was not noticeable in the disc proper (**D**), although characteristic staining of subretinal glial cells was observed (not shown). *Src42A^GS^* (arrowhead, **E**) or *Src64B* (arrowhead, **F**) mosaic eye discs showed upregulation of βgal protein representing *msn-lacZ* reporter expression. Smaller clones in *Src42A^GS^* (arrow, **Ei**) or *Src64B* (arrow, **Fi**) mosaic eye discs did not show upregulation of the *msn-lacZ* reporter.

Src-expressing mosaic eye discs were assessed for alterations to apoptosis by terminal deoxynucleotidyl transferase dUTP nick end labelling (TUNEL) assays. In comparison to control mosaic discs (Figure 5A), expression of *Src42A^GS^* (Figure 5B) or *Src64B* (Figure 5C) resulted in a higher number of TUNEL-positive cells that appeared both within and around the clones (Figure 5Bi,Ci, arrowheads). Thus, the expression of *Src42A^GS^* or *Src64B* in mosaic eye discs resulted in induction of cell death. Since no discernible effect on S phases were observed in mosaic eye discs expressing *Src42A^GS^* or *Src64B*, it is likely that the reduced clonal size of *Src*-expressing clones in the eye disc is due to an increase in cell death rather than an inhibition of cell proliferation.

To assess the consequences of blocking cell death in Src-expressing clones, the *p35* caspase inhibitor transgene [68] was coexpressed with *Src42A^GS^* or *Src64B* in eye disc clones and the effect on clonal tissue size, differentiation and F-actin organisation was examined. *Src42A^GS^ + p35* clonal tissue size (Figure S5C and Table 2) was only marginally larger than *Src42A^GS^*-expressing clones (Figure S5B) and remained smaller than control (Figure 2A,B) or *p35*-expressing clones (Figure S5A). Clonal expression of *Src42A^GS^ + p35* in clones disrupted the overall morphology of eye tissue resulting in an eye disc that was undulated (Figure S5C) compared with the wild-type control (Figure 2A,B), *p35* (Figure S5A) or *Src42A^GS^* mosaic eye discs (Figure S5B). This change in tissue morphology correlated with the disrupted patterning of differentiating cells. F-actin staining showed that it was the wild-type tissue, rather than *Src42A^GS^ + p35* clones that was overgrown and folded (Figure S5C), suggesting non-cell-autonomous overgrowth effects. F-actin was enriched in *Src42A^GS^ + p35* clones (Figure S5C). Similar effects were observed for *Src64B + p35* mosaic eye discs: *Src64B + p35* clonal tissue (Figure S5E and Table 3) was not noticeably larger than that of *Src64B*-expression alone (Figure S5D), but the pattern of differentiation was disrupted and the morphology of the eye disc was no longer planar, due to the distortion of the surrounding wild-type tissue (Figure S5E), suggesting non-cell autonomous tissue growth. This non-cell autonomous effect is similar to that observed by “undead” cells, where apoptosis is initiated but prevented by p35 expression, leading to the secretion of morphogens and non-cell autonomous proliferation [80].

Indeed, BrdU incorporation assays showed that coexpression of *Src42A^GS^ + p35* (Figure S5H and Table 2) or *Src64B + p35* in clones (Figure S5J and Table 3) resulted in an increase in S phases in the surrounding wild-type tissue. S phases were generally not observed in clones expressing *Src42A + p35* (Figure S5H) or *Src64B + p35* (Figure S5J), relative to *p35*-expressing control eye epithelium (Figure S5F). In comparison with the normal *Src42A^GS^* mosaic adult eyes (Figure S5L), *Src42A^GS^ + p35* mosaic adult eyes were folded and overgrown (Figure S5M and Table 2), reflecting the ectopic proliferation observed during third instar. Whilst expression of *Src64B* resulted in adult flies with normal eyes (Figure S5N), *Src64B + p35* expression in clones resulted in lethality during the third instar larval stage (Figure S5O and Table 3), suggesting that uncontrolled cell proliferation might have impaired metamorphosis.

### 2.8. Expression of Src in Eye Disc Clones Promotes JNK Pathway Signalling and Activity

It has been shown that Src regulates JNK signalling during dorsal closure [50] and activation of Src, indirectly via loss of Csk, requires JNK signalling in cell proliferation, apoptosis and cell migration [40,47]. JNK signalling has also been shown to mediate cell death signals in both mammalian and *Drosophila* studies [81,82,83]. Thus, to determine if JNK signalling was activated in Src-expressing clones in the eye epithelium, JNK pathway activity was assessed using the *misshapen-lacZ* (*msn-lacZ*) JNK pathway enhancer trap [84]. In control mosaic eye discs, JNK pathway activation reported by *msn-lacZ* was undetectable (Figure 5D). Expression of *Src42A^GS^* (Figure 5E, arrowhead) or *Src64B* (Figure 5F, arrowhead) in clones resulted in upregulation of *msn-lacZ* in clonal tissue, indicating that the JNK pathway was activated. However, smaller clones in *Src42A^GS^*- (Figure 5Ei, arrow) or *Src64B*- (Figure 5Fi, arrow) expressing mosaic eye discs did not show upregulation of the *msn-lacZ* reporter. Thus, JNK pathway activity is activated in larger Src-expressing clones that lose cell polarity (Figure S4).

To more directly assess JNK activation, Src-expressing mosaic eye discs were stained with a mammalian phospho-specific JNK (pJNK) antibody that cross-reacts with *Drosophila* JNK [50,85,86]. Compared with the low pJNK signal in control mosaic eye discs (Figure S6A), *Src42A^GS^*-expressing clones showed an increase in pJNK signal (Figure S6B, arrowhead, with coexpression of *p35* to increase clonal tissue size). Similarly, *Src64B*-expressing mosaic eye discs showed a strong upregulation of pJNK in clonal tissue (Figure S6C, arrowhead) in comparison to control mosaic eye antennal discs (Figure S6A). As observed with the *msn-lacZ* reporter (Figure 5E,F), pJNK was not discernible in small clones of either *Src42A^GS^ + p35-* or *Src64B*-expressing mosaic eye discs ((Figure S6Bi,Ci) arrow), although it was detected in large *Src42A^GS^ + p35-* or *Src64B*-expressing clones (Figure S6Bi,Ci and Table 2 and Table 3).

### 2.9. Blocking JNK Increases Clone Viability of Src-Expressing Clones, Reduces F-actin Accumulation and Results in Basal Extrusion

**Figure 6:**
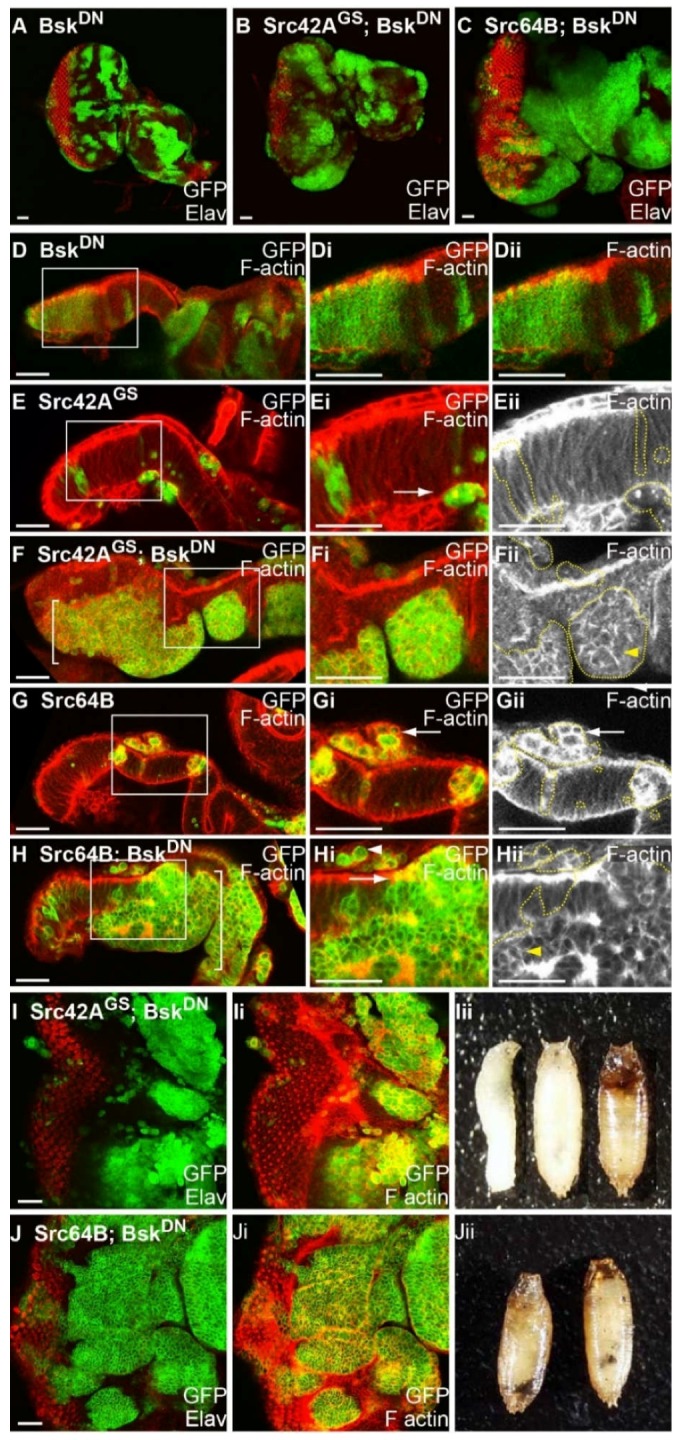
Blocking JNK results in increased clone viability and basally localised clonal tissue. Confocal images, planar views or cross-sections, of eye-antennal discs: Clones are marked by GFP (green in merged images). Elav marks differentiated cells (red in merged images **A**–**C**,**I**,**J**) and rhodamine-phalloidin visualises F-actin to mark cell outlines (in **D**–**H** as marked, red in merged images, **ii**; red in merged images, **Ii**,**Ji**). Light micrographs **Iii**,**Jii**. (**A**–**D**) Planar views (**E**–**H**) Cross-sections, (**I**,**J**) Planar views scale bar, 25 μM. Genotypes: (**A**,**D**) Bsk^DN^: *ey-FLP1*, *UAS-mCD8-GFP/+; +/+; tub-GAL4 FRT82B tub-GAL80/FRT82B UAS-bsk^DN^*; (**B**,**F**,**J**) Src42A^GS^; Bsk^DN^: *ey-FLP1*, *UAS-mCD8-GFP/+; GS11049/+*; *tub-GAL4 FRT82B tub-GAL80/FRT82B UAS-bsk^DN^*; (**C**,**H**,**I**) Src64B; Bsk^DN^: *ey-FLP1*, *UAS-mCD8-GFP/+; UAS-Src64B/+; tub-GAL4 FRT82B tub-GAL80/FRT82B UAS-bsk^DN^* (**E**) Src42A^GS^: *ey-FLP1*, *UAS-mCD8-GFP/+; GS11049*/+*; tub-GAL4 FRT82B tub-GAL80/FRT82B*; and (**G**) Src64B: *ey-FLP1*, *UAS-mCD8-GFP/+; UAS-Src64B/+; tub-GAL4 FRT82B tub-GAL80/FRT82B*. Eye clones expressing *bsk^DN^* demonstrated similar patterns of differentiation (red, **A**) compared to wild-type controls (Figure 2A). Expression of *bsk^DN^* in *Src42A^GS^* (**B**) or *Src64B* (**C**) clones resulted in increased clone viability compared with expression of *Src42A^GS^* (Figure 2E) or *Src64B* alone (Figure 2I). Cross sections indicate F-actin was enriched at the apical surface in *bsk^DN^* mosaic eye discs (**E**), comparable to control mosaic eye discs (Figure 4C). Expression of *Src42A^GS^* in mosaic eye discs (**F**) resulted in reduced-sized clones that localised to the basal part of the epithelium (arrow, **Ei**). In comparison, coexpression of *Src42A^GS^ + Bsk^DN^* resulted in increased clonal tissue size (**F**). These clones had smooth borders and localised in the basal part of the epithelium (bracket, **F**). Smaller clones were observed within the epithelium and above the apical surface (arrowhead, **Fii**). Cells within the clones were rounded, as outlined by membrane bound GFP (**Fi**,**Fii**). F-actin was cortically enriched in the centre of large clonal clusters (yellow arrowhead, **Fii**). *Src64B*-expressing clones form rounded, discrete clusters (arrow, **Gi**,**Gii**) that were enriched for F-actin (arrow, **Gi**,**Gii**) in comparison with surrounding wild-type tissue. Cells within clones were rounded and outlined with F-actin (**Gii**). Coexpression of *Src64B + bsk^DN^* (**H**) resulted in larger clones that were basally localised (bracket, **H**). Smaller clones were also observed just above the apical surface of the eye disc (white arrowhead, **Hi**). In clones that immediately abut wild-type tissue, clonal cells had apically enriched F-actin (white arrow, **Hi**) similar to that observed in adjacent wild-type tissue. F-actin staining outlines the cell cortex (yellow arrow, **Hii**). In *Src42A^GS^ + bsk^DN^* (**I**) and *Src64B + bsk^DN^* (**J**) mosaic eye discs, differentiation occurred, although the patterning was disrupted (**I**,**J**). F-actin was increased in clones and outlined cells within clonal tissue (**Ii**,**Ji**) and lethality occurred during late larval/pupal stages (**Iii**,**Jii**); melanotic masses were observed in *Src64B + bsk^DN^* pupae (pupae on right, **Jii**).

Since the JNK pathway is activated in Src-expressing clones, we used a dominant negative, kinase dead, form of *Drosophila* JNK *basket*, (*bsk^DN^*) [87], to investigate whether blocking JNK signalling could suppress the Src overexpression clonal phenotype. Bsk^DN^ has been used extensively to inhibit JNK pathway signalling, and functions similarly or more potently than loss-of-function alleles or other approaches to reduce JNK activity [51,71,88,89,90]. In comparison to the control (Figure 2A,B), mosaic eye discs expressing *bsk^DN^* showed no discernible effects on differentiation (Figure 6A) or actin organisation (Figure 6D) in apical or basal sections during larval eye development, and *bsk^DN^*-expressing mosaic adult eyes were comparable to control adult eyes. To test whether loss of JNK could suppress Src clonal phenotypes, *Src42A^GS^* or *Src64B* were coexpressed with *bsk^DN^* in mosaic eye discs and examined for alterations in clone size, and markers of differentiation (Elav) and F-actin organisation (phalloidin). Expression of *bsk^DN^* in *Src42A^GS^-* (Figure 6B and Table 2) or *Src64B-* (Figure 6C and Table 3) expressing clones resulted in increased clone viability compared with clonal expression of *Src42A^GS^* (Figure 2E,F) or *Src64B* (Figure 2I,J) alone. Cross-sections of clones coexpressing *Src42A^GS^ + bsk^DN^* (Figure 6F) or *Src64B + bsk^DN^* (Figure 6H) revealed that clonal tissue was dramatically increased in size and localised to the basal part of the epithelium compared to *Src42A^GS^*- or *Src64B*-expressed alone in mosaic eye discs, which showed only small *Src42A^GS^*-expressing clones residing in the basal part of the epithelium (Figure 6E,G). These large clonal clusters were characterised by smooth borders and contained rounded cells (Figure 6F,H), with enrichment of cortical F-actin in the large clonal clusters (Figure 6Fii,Hii, yellow arrowheads), relative to *bsk^DN^* control eye discs (Figure 6D). Additionally, smaller clones of *Src42A^GS^*- or *Src64B*- + *bsk^DN^*-expressing cells were observed within the epithelium and above the apical surface of the epithelium (Figure 6F,H), as were also observed with *Src42A^GS^*- or *Src64B*-expressing cells (Figure 6E,G). Cells in *Src64B*-expressing clones showed an enrichment of F-actin around the cell cortex (Figure 6Gi,Gii, arrow), whereas F-actin in *Src64B* + *bsk^DN^* cells appear to have reduced cortical staining (Figure 6Gi, arrow). Further, in regions of the larger *Src64B* + *bsk^DN^* clones that border wild-type tissue, F-actin appears to be apically enriched similar to that of adjacent wild-type cells (Figure 6Hi, white arrowhead). These observations suggest that Src64B-mediated JNK activation may normally promote F-actin polymerisation leading to enriched F-actin in clonal tissue. Although patterning was disrupted, differentiated cells were still observed in *Src42A^GS^ + bsk^DN^* (Figure 6I) and *Src64B + bsk^DN^* mosaic eye discs (Figure 6J), however, the *Src + bsk^DN^* larva did not develop to adulthood, with lethality occurring during late larval/pupal stages (Figure 6Iii,Jii). Black melanotic masses were sometimes observed in *Src64B + bsk^DN^* pupae (Figure 6Jii and Table 3). Altogether, these data show that blocking the JNK pathway increases viability of Src-expressing clones and suppresses F-actin accumulation, suggesting that JNK acts downstream of Src to induce cell death and F-actin polymerisation consistent with other studies in the *Drosophila* wing epithelium [88,89], and eye epithelium [44]. 

### 2.10. JNK is Activated in Src + Ras^ACT^ Neoplastic Overgrowth, and Blocking JNK Results in Partial Suppression of the Overgrowth and Differentiation Defects

**Figure 7:**
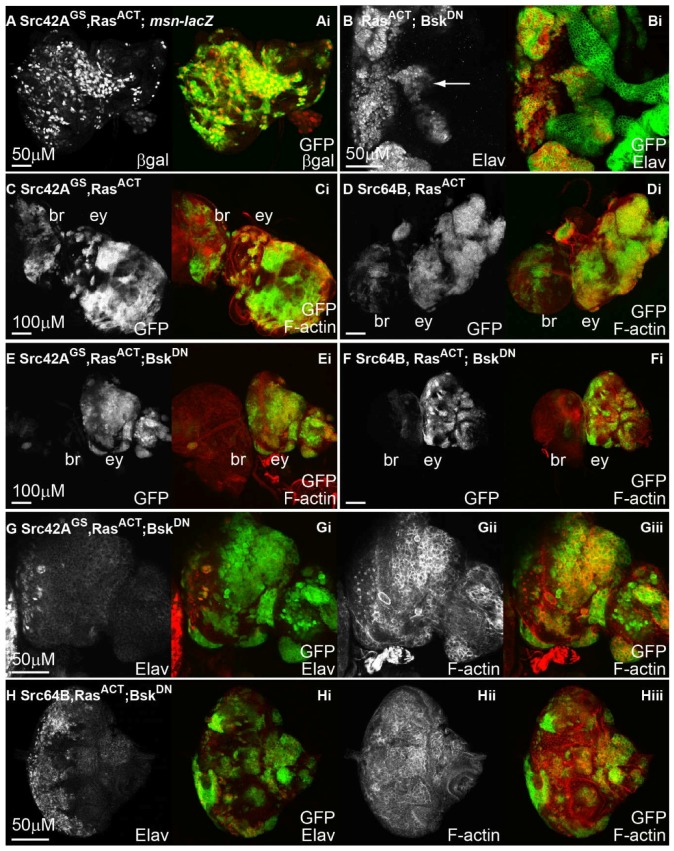
JNK pathway is activated in *Src + Ras^ACT^* eye disc clones, and blocking JNK signalling in *Src + Ras^ACT^* clones suppresses clonal tissue overgrowth and restores differentiation. Confocal images, planar views, of eye-antennal discs. Clones are marked by expression of GFP (green in merged images). βgal antibody detection measures transcription of the *msn-lacZ* enhancer trap (red in merged images). Elav marks differentiated cells (in **B**,**G**,**H** as marked, red in merged images, **i**) and rhodamine-phalloidin visualises F-actin to mark cell outlines (in **C**–**H** as marked, red in merged images, **Ci**–**Fi** and **Giii**,**Hiii**). (**A**) Scale bar, 50 μM; (**B**–**F**) Scale bar, 100 μM; and (**G**,**H**) Scale bar, 50 μM. Genotypes: (**A**) Src42A^GS^, Ras^ACT^; *msn-lacZ*: *ey-FLP1*, *UAS-mCD8-GFP/+; GS11049*, *UAS-Ras^ACT^/+; tub-GAL4 FRT82B tub-GAL80/msn-lacZ FRT82B*; (**B**) Ras^ACT^; Bsk^DN^: *ey-FLP1*, *UAS-mCD8-GFP/+; UAS-Ras^ACT^/+; tub-GAL4 FRT82B tub-GAL80/FRT82B UAS-bsk^DN^*; (**C**) Src42A^GS^, Ras^ACT^: *ey-FLP1*, *UAS-mCD8-GFP/+; GS11049*, *UAS-Ras^ACT^/+; tub-GAL4 FRT82B tub-GAL80/FRT82B*; (**D**) Src64B, Ras^ACT^: *ey-FLP1*, *UAS-mCD8-GFP/+; UAS-Src64B*, *UAS-Ras^ACT^/+; tub-GAL4 FRT82B tub-GAL80/FRT82B*; (**E**,**G**) Src42A^GS^, Ras^ACT^; Bsk^DN^: *ey-FLP1*, *UAS-mCD8-GFP/+; GS11049*, *UAS-Ras^ACT^/+; tub-GAL4 FRT82B tub-GAL80/FRT82B UAS-bsk^DN^*; and (**F**,**H**) Src64B, Ras^ACT^; Bsk^DN^: *ey-FLP1*, *UAS-mCD8-GFP/+; UAS-Src64B*, *UAS-Ras^ACT^/+; tub-GAL4 FRT82B tub-GAL80/FRT82B UAS-bsk^DN^. Src42A^GS^ + Ras^ACT^* clones show upregulation of the *msn-lacZ* reporter (**A**). Expression of *Ras^ACT^ + bsk^DN^* in clones (**B**) resulted in ectopic differentiation anterior to the morphogenetic furrow (white arrow, **B**), comparable to that of expression of *Ras^ACT^* alone (Figure 2C). Expression of *Src42A^GS^ + Ras^ACT^* (**C**) or *Src64B + Ras^ACT^* (**D**) in mosaic eye discs resulted in tissue overgrowth with indistinguishable eye or antennal tissue. Coexpression of *bsk^DN^* with *Src42A^GS^ + Ras^ACT^* (**E**) or *Src64B + Ras^ACT^* (**F**) resulted in partial suppression of overgrowth, and eye imaginal discs regained normal shape and morphology. Protrusive clones were not observed within brain lobes. Differentiation defects observed in *Src42A^GS^ + Ras^ACT^* clones (Figure 2G) and *Src64B + Ras^ACT^* eye disc clones (Figure 2K) were suppressed upon expression of *bsk^DN^* (**G**,**H**, respectively). Differentiation occurred in both wild-type and clonal tissue (**Gi**,**Hi**), although as indicated by the gaps in staining, some cells were incorrectly localised basally. Tissue morphology was disrupted and F-actin was increased in clonal tissue (**Gii**,**Giii**,**Hii**,**Hiii**).

Given the requirement for JNK signalling in Src-expressing clones shown here and previously [50], and the role of JNK signalling in cooperative interactions observed between cell polarity mutants and Ras-driven tumourigenesis [51,71,74,90,91,92,93,94,95,96], it is conceivable that JNK activity could be upregulated and/or required for the cooperative overgrowth between *Src + Ras^ACT^*-induced neoplastic overgrowth. Therefore, to assess whether JNK signalling was active in *Src + Ras^ACT^* clonal tissue, the *msn-lacZ* reporter was utilised as a readout for JNK pathway activation. Compared with control and *Src42A^GS^* mosaic eye discs (Figure 5D–F), expression of *Src42A^GS^ + Ras^ACT^* in eye disc clones results in upregulation of the *msn-lacZ* reporter within most clonal cells (Figure 7A and Table 2). Thus, JNK signalling is upregulated in *Src* and *Ras^ACT^* expressing tissue, consistent with it also playing a role in tumourigenesis in this setting.

To determine whether blocking the JNK pathway could alter *Src + Ras^ACT^* clonal neoplastic overgrowth, *bsk^DN^* was coexpressed in *Src + Ras^ACT^* eye disc clones, and mosaic eye discs were analysed for changes in clonal tissue size (marked by GFP expression), cell morphology (F-actin marked by phalloidin) and differentiation (marked by Elav). Expression of *bsk^DN^* in *Ras^ACT^*-expressing clones (Figure 7B) resulted in ectopic differentiation, similar to that observed upon expression of *Ras^ACT^* alone (Figure 2C,D). Similar to that observed for *Ras^ACT^* mosaic eye discs, expression of *Ras^ACT^ + bsk^DN^* resulted in pupal lethality. Whereas *Src42A^GS^ + Ras^ACT^* (Figure 7C) or *Src64B + Ras^ACT^* (Figure 7D) expression in mosaic eye discs resulted in dramatic tissue overgrowth, striking suppression of overgrowth was observed upon blocking JNK signalling by coexpression of *bsk^DN^* with *Src42A^GS^ + Ras^ACT^* (Figure 7E and Table 2) or *Src64B + Ras^ACT^* (Figure 7F and Table 3) in mosaic eye discs. The eye antennal disc had normal tissue morphology with recognisable shape and structure (Figure 7E,F). Higher magnification views revealed that F-actin was enriched cortically in clonal tissue and that tissue morphology of the eye disc was still disrupted (Figure 7G,Hii,Hiii). In contrast to *Src* overexpression with *Ras^ACT^*, where clonal tissue was observed in the brain lobes (Figure 3C,E), co-expression of *bsk^DN^* resulted in reduced clonal tissue observed in the brain lobes of *Src + Ras^ACT^* mosaic larvae (Figure 7E,F). Whilst *Src42A^GS^ + Ras^ACT^* clones exhibited protrusive morphology (Figure 3C), this was no longer observed upon *bsk^DN^* expression (Figure 7E and Table 2). Moreover, the loss of differentiation observed in *Src + Ras^ACT^* mosaic eye discs (Figure 2G,H,K,L) was partially suppressed when JNK was blocked by expression of *bsk^DN^* (Figure 7G,H and Table 2). Differentiation occurred in both wild-type and *Src + Ras^ACT^ + bsk^DN^* clonal tissue (Figure 7G,Hi), although some cells were incorrectly basally localised. However, in comparison to control mosaic eye discs (Figure 4A,B), differentiation was not restored completely to normal in *Src + Ras^ACT^ + bsk^DN^* mosaic eye discs. Thus, expression of *Src + Ras^ACT^* in eye disc clones results in robust upregulation of JNK pathway (as measured by JNK pathway enhancer trap, *msn-lacZ*), and blocking the JNK pathway partially suppressed tissue overgrowth of the eye-antennal disc, restored the morphology of the eye-antennal disc and partially restored differentiation to the *Src + Ras^ACT^*-expressing clones. Altogether, these results show that JNK activation is required for *Src + Ras^ACT^* cooperative, neoplastic overgrowth.

### 2.11. Ras-Raf-MAPK and Ras-PI3K Pathways are Required with Src for Cooperative Tumourigenesis

Ras conveys its signals by many effectors, of which the most well-known are Raf-mitogen-activated protein kinase (MAPK), Phosphoinositide 3-Kinase (PI3K) and Ral pathways [23,97], however in *Drosophila*, *Ras^ACT^*-mediated tissue growth effects were mediated by Raf-MAPK and PI3K (Dp110/PI3K92E) effectors [62,64,98]. In cooperation with *scrib* mutant however, only activated *Raf* (*Raf^GOF^*) [99] is able to phenocopy the effects of *Ras* function to result in neoplastic overgrowth [72,100]. This requirement of Raf-MAPK is likely to be specific as expression of other Ras effectors PI3K or Ral were unable to recapitulate the effects of *Ras^ACT^* in *scrib* mutant clones [72].

**Figure 8:**
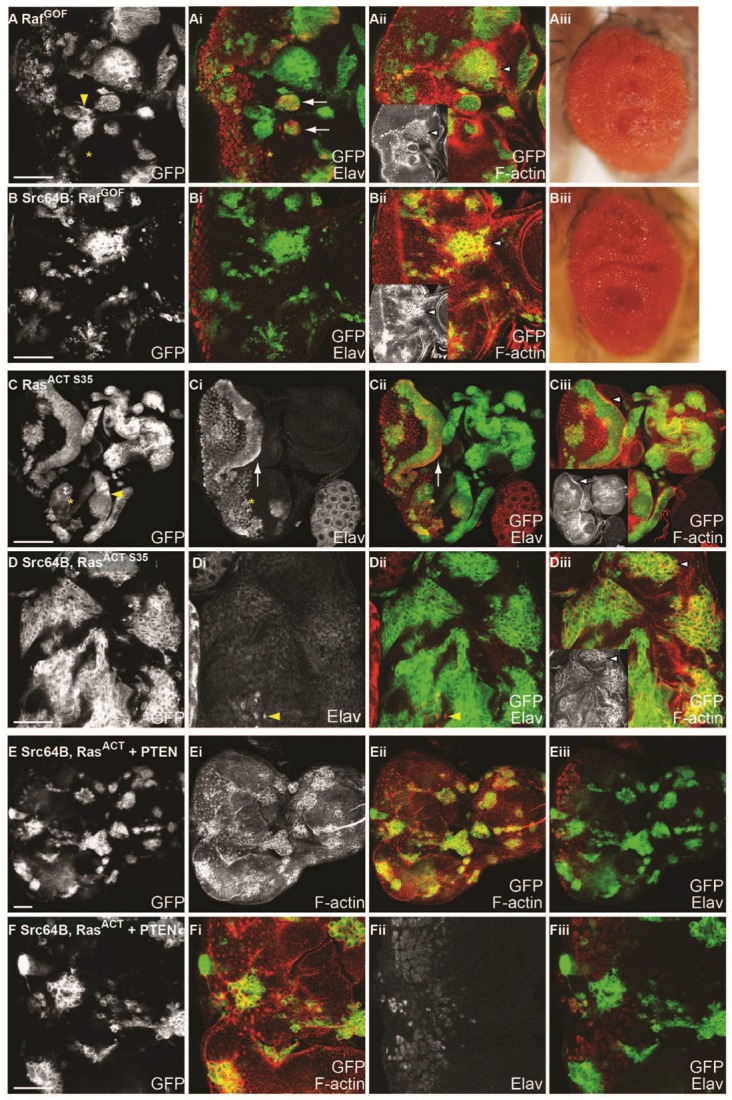
*Src + Ras^ACT^* cooperative overgrowth requires the contribution of Raf and PI3K signalling. Planar confocal images of eye-antennal discs. Clones are marked by expression of GFP (green in merged images). Asterisk marks the morphogenetic furrow, Elav marks differentiated cells (in **A**–**F** as marked, red in merged images: **Ai**,**Bi**,**Cii**,**Dii**,**Eiii,Fiii**) and rhodamine-phalloidin visualises F-actin to mark cell outlines (in **A**–**F** as marked, red in merged images, **Aii,Bii**,**Ciii,Diii**,**Eii**,**Fi** and grey in insets of **Aii**,**Bii**,**Ciii** and **Diii**). Scale bar, 50 μM. Genotypes: (**A**) Raf^GOF^: *ey-FLP1*, *UAS-mCD8-GFP/+; +/+; tub-GAL4 FRT82B tub-GAL80/FRT82B UAS-Raf^GOF^*; (**B**) Src64B; Raf^GOF^: *ey-FLP1*, *UAS-mCD8-GFP/+; UAS-Src64B/+; tub-GAL4 FRT82B tub-GAL80/FRT82B UAS-Raf^GOF^*; (**C**) Ras^ACT S35^: *ey-FLP1*, *UAS-mCD8-GFP/+; +/+; tub-GAL4 FRT82B tub-GAL80/FRT82B UAS-Ras^ACT S35^*; (**D**) Src64B; Ras^ACT S35^: *ey-FLP1*, *UAS-mCD8-GFP/+; UAS-Src64B/+; tub-GAL4 FRT82B tub-GAL80/FRT82B UAS-Ras^ACT S35^*; and (**E**,**F**) *Src64B*, *Ras^ACT^/PTEN*: *ey-FLP1*, *UAS-mCD8-GFP/+; UAS-Src64B*, *UAS-Ras^ACT^/UAS-PTEN; tub-GAL4 FRT82B tub-GAL80/FRT82B.* In control mosaic eye discs, differentiated cells were restricted to the posterior region of the eye (refer to Figure 4A). Expression of *Raf^GOF^* (**A**) resulted in ectopic differentiation (arrow, **Ai**) in rounded clones (yellow arrowhead) anterior to the morphogenetic furrow (asterisk). The resulting adult eyes had a folded, slightly overgrown phenotype (**Aiii**). Coexpression of *Src64B + Raf^GOF^* (**B**) produced some rounded clones with reduced differentiation (**Bi**). Compared to *Raf^GOF^* alone (**A**), no ectopic differentiation was observed in *Src64B + Raf^GOF^* clones (**Bi**) and clonal tissue were smaller in size compared to *Raf^GOF^* (**A**). F-actin was enriched in *Src64B + Raf^GOF^* clones (**Bii**, arrowhead) compared to clones expressing *Raf^GOF^* alone (**Aii**, arrowhead). *Src64B + Raf^GOF^* (**Biii**) adult eyes had a rough and folded phenotype comparable to *Raf^GOF^* adult eyes (**Aiii**). Expression of *Ras^ACT-S35^* in mosaic eye discs resulted in ectopic differentiation (arrow, **Ci,Cii**) in clones immediately anterior to the morphogenetic furrow (asterisk) as observed in *Ras^ACT^* mosaic eye discs (Figure 2C). *Ras^ACT-S35^* expressing ells are abnormally arranged due to tissue misfolding, but F-actin appears to be apically localised (white arrowhead, **C**, **Ciii** and inset). Expression of *Ras^ACT-S35^* resulted in pupal lethality. Coexpression of *Src64B + Ras^ACT-S35^* (**D**) resulted in tissue overgrowth where GFP-marked clonal tissue represented more than half of the eye disc. There was a general loss of differentiation in mosaic eye discs expressing *Src64B + Ras^ACT-S35^* (**Di**), although a very small number of cells were observed to differentiate (yellow arrowhead, **Di**,**Dii**). Actin organisation was disrupted and F-actin levels were increased within clonal tissue (white arrowhead, **Diii** and inset). Expression of *Src64B + Ras^ACT^* in eye disc clones resulted in over-representation of GFP-marked clonal tissue (Figure 2K,L). Coexpression of *PTEN* in *Src64B + Ras^ACT^* eye disc clones resulted in reduced GFP-marked clonal tissue (**E**), which was enriched for F-actin compared with surrounding wild-type tissue (**Ei,Eii,Fi**), although differentiation was not restored (**Eiii,Fii,Fiii**).

To test whether Src kinases similarly cooperate with Raf signalling in clonal analysis, *Src42A^GS^* or *Src64B* were coexpressed with an amino-terminal truncation allele of Raf, *Raf^GOF^*, which renders constitutive activation of Raf signalling [99]. As observed in *Ras^ACT^* mosaic eye discs, expression of *Raf^GOF^* resulted in rounded clones with smooth borders and precocious differentiation anterior to the morphogenetic furrow (Figure 8Ai). *Raf^GOF^* mosaic eye discs exhibited disrupted tissue morphology resulting in an enrichment of F-actin at the borders between wild-type and mutant clonal tissue (Figure 8Aii). Unlike *Ras^ACT^*, clonal expression of *Raf^GOF^* did not lead to pupal lethality but resulted in adult flies with folded, overgrown eye tissue (Figure 8Aiii). To determine whether Raf signalling could phenocopy *Ras^ACT^* in cooperation with *Drosophila* Src kinases, *Raf^GOF^* was coexpressed with *Src42A* or *Src64B* in mosaic eye discs. Surprisingly, cooperative overgrowth was not observed; instead, expression of *Src42A^GS^ + Raf^GOF^* or *Src64B + Raf^GOF^* (Figure 8Bi and Table 4) resulted in rounded clones that did not differentiate, although some clones appear smaller than that of clones expressing *Raf^GOF^* alone. Thus, although Src expression inhibits Raf-induced differentiation, *Src* was unable to cooperate with *Raf^GOF^*. F-actin was enriched in *Src64B + Raf^GOF^* clonal tissue (Figure 8Bii and inset, arrowhead) compared to clones expressing *Raf^GOF^* alone (Figure 8Aii and inset, arrowhead). Adult eyes expressing *Src42A^GS^ + Raf^GOF^* or *Src64B + Raf^GOF^* (Figure 8Biii) were rough and folded, comparable to the hyperplastic *Raf^GOF^* adult eye phenotype (Figure 8Aiii). Thus, these data suggest that Raf signalling is not sufficient to phenocopy Ras in cooperation with Src family kinases, and other Ras effectors may be required for neoplastic overgrowth.

Since activation of the Raf-MAPK cascade alone was not sufficient to cooperate with Src family kinases, the *Ras^ACT-S35^* effector domain mutant [62] was utilised to test the contribution of Raf signalling. The *Ras^ACT-S35^* mutant preferentially signals to the Raf-MAPK pathway and has been characterised in both mammals and *Drosophila* [61,62,64,101]. Specifically, previous analysis in *Drosophila* eye and wing discs has demonstrated that *Ras^ACT-S35^* favours Raf-MAPK and is less potent than *Ras^ACT^* in recruitment of a PI3K reporter [62,64]. Firstly, we tested the effect of expression of *Ras^ACT-S35^* alone in mosaic eye discs by examining differentiation and F-actin organisation. *Ras^ACT-S35^* mosaic eye discs were characterised by rounded clones with smooth borders (Figure 8C, yellow arrowhead) and ectopic differentiation anterior to the morphogenetic furrow (Figure 8C, asterisk). Due to tissue misfolding of *Ras^ACT-S35^* mosaic eye tissue, cells are abnormally arranged, but F-actin appears to be apically localised (Figure 8Ciii and inset, white arrowhead). These effects were similar to that observed in *Ras^ACT^* mosaic eye discs (Figure 2B), and, like *Ras^ACT^*, expression of *Ras^ACT-S35^* resulted in pupal lethality.

To determine whether *Src* expression could cooperate with *Ras^ACT-S35^*, *Src42A^GS^* or *Src64B* were coexpressed with *Ras^ACT-S35^* in mosaic eye discs. Expression of *Src42A^GS^ + Ras^ACT-S35^* or *Src64B + Ras^ACT-S35^* (Figure 8D) in mosaic eye discs resulted in overgrowth of clonal tissue (Figure 8D, GFP-marked, and Table 4). There was a loss of differentiation in both wild-type and clonal tissue, although on rare occasions, differentiation occurred in wild-type tissue (Figure 8Di,Dii, yellow arrowhead). F-actin was enriched within clonal tissue and F-actin organisation was disrupted (Figure 8Diii and inset, white arrowhead). Distinct from *Src64B + Ras^ACT^*-expressing mosaic eye discs where clonal tissue largely predominates in the eye disc (Figure 2K,L), *Src64B + Ras^ACT-S35^* clonal tissue, while still over-represented, did not overtake the whole eye disc (Figure 8D). This suggested that *Ras^ACT-S35^* is less potent than *Ras^ACT^* in cooperation with *Src*. Thus, while expression of activated Raf was not sufficient for cooperation with Src, expression of *Ras^ACT-S35^*, which preferentially but not exclusively signals via Raf, was able to cooperate with *Src*, albeit to a lesser extent than *Ras^ACT^*. These data suggest that Raf signalling is required for neoplastic overgrowth in concert with Src overexpression, however, other Ras effectors are also likely to be required.

To test the whether the PI3K pathway, an effector of *Ras^ACT^* in tissue growth control [64], was required for *Src + Ras^ACT^* tumourigenesis, we co-expressed PTEN (which antagonises the activity of PI3K) or a dominant-negative version of PI3K (*Dp110^DN^*) in *Src64B +Ras^ACT^* clones in the eye-antennal epithelium (Figure 8E,F and Figure S7). Expression of PTEN alone did not affect the clone size (Figure S7A) compared to wild-type (Figure 2A,B). However, expression of *PTEN* with *Ras^ACT^* or *Src64B* (Figure S7B,C) mildly reduced clonal size relative to *Ras^ACT^* or *Src64B* alone (Figure 2C,D,I,J). Strikingly, co-expression of *PTEN* in *Src64B* + *Ras^ACT^* eye disc clones dramatically reduced tumour growth and restored differentiation in most clones (Figure 8E,F and Table 4), compared to *Src64B* + *Ras^ACT^* tumours (Figure 2K,L). To more directly assess the requirement of PI3K activity, we co-expressed of a dominant negative allele of the *Phosphoinositide 3-Kinase* PI3K gene, *Dp110*/*PI3K92E*(*Dp110^DN^*) in *Src64B* + *Ras^ACT^* eye disc clones also reduced tumour size (Figure S7G,H and Table 4), although not as potently as with *PTEN* (Figure 8E,F). Thus, consistent with the data showing that *Raf^GOF^* expression alone were insufficient to cooperate with Src (Figure 8A,B), we show that PI3K activity plays an important function in *Src64B* + *Ras^ACT^* tumourigenesis.

## 3. Discussion

**Figure 9:**
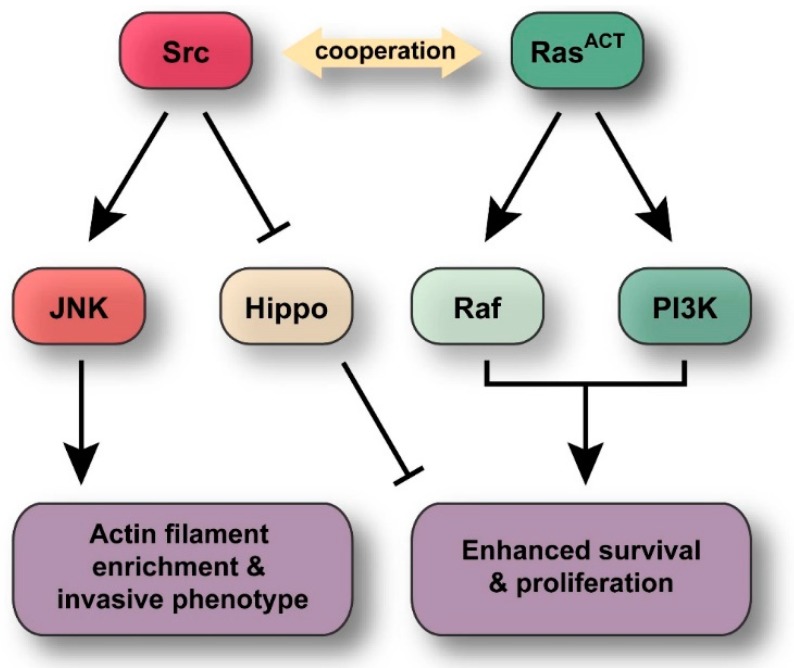
Model for the cooperation of Src and oncogenic Ras in tumourigenesis. Coexpression of Src and oncogenic Ras (Ras^V12/ACT^) results in cooperative tumourigenesis in *Drosophila* epithelia, resulting in invasive, overgrown tumours characterised by loss of differentiation and F-actin accumulation. Upon Src expression, JNK pathway signalling is activated and the Hippo pathway is inhibited, whilst downstream of oncogenic Ras, activation of Raf and PI3K is required for cooperative tumourigenesis with Src.

In this study, we have identified the *Drosophila* Src kinase genes, *Src42A* and *Src64B*, as cooperating genes with activated Ras in tumourigenesis in the eye-antennal epithelial tissue. *Src42A* and *Src64B* were identified in a genetic screen as enhancers of the *ey > Ras^ACT^* hyperplastic adult eye phenotype. Importantly, in a clonal context, the *Drosophila* Src kinases were also able to cooperate with *Ras^ACT^* resulting in neoplastic overgrowth of the eye-antennal epithelium (Figure 9). This cooperative tumourigenesis is characterised by tissue overgrowth, increased clonal tissue, loss of differentiation, disruption to F-actin organisation, larval lethality and an invasive clonal phenotype. Src expressed alone in eye disc clones results in increased apoptosis, a loss of cell polarity and disruption to F-actin organisation, but was not sufficient alone to promote tumour formation. We show that JNK signalling acts downstream of Src to promote cell death and increased accumulation of F-actin. In the context of *Src + Ras^ACT^* tumours, the JNK pathway contributes to the inhibition of differentiation, clonal overgrowth and invasive phenotypes associated with *Src + Ras^ACT^* neoplastic overgrowth. Moreover, we show that the PI3K pathway is critical for the cooperation of *Src* with *Ras^ACT^* in tumourigenesis. Altogether, our findings provide insight into the mechanism by which Src and Ras signalling cooperate in tumourigenesis, which may provide new avenues for the treatment of human cancer.

Others have provided indirect evidence that Src signalling can cooperate with *Ras^ACT^* in a clonal context [39]. However, in this previous study, *Ras^ACT^* was expressed in *Csk*-deficient clones, mimicking Src activation, to result in tissue overgrowth and delayed development [39]. Since it is unclear whether Csk may act solely through Src kinases [41], our study has provided evidence that *Src* is able to cooperate with *Ras^ACT^* in tumourigenesis in the eye-antennal epithelium. Furthermore, additional expression of *Src64B* with *Ras^ACT^* in *Csk*-mutant clones resulted in an enhancement of this phenotype [39]. This correlates with our observations using the *GMR-GAL4* system that Src expression can elicit a dose-dependent response. Although expression of all three *Src42A* overexpression lines tested cooperated with *Ras^ACT^*, there were differences observed. There was little differentiation observed in *Src42A^ACT^ + Ras^ACT^* mosaic eye discs, whereas expression of wild type *Src42A + Ras^ACT^* enabled some differentiation, albeit aberrantly, to occur in the mosaic eye disc.

While Src is known to affect many proteins that modulate actin dynamics [44,88,89,102,103], blocking JNK in Src-expressing clones suppresses the F-actin enrichment that is associated with Src-expressed alone. This shows that Src may also act via JNK, at least in part, to mediate changes in F-actin organization. In fact, JNK upregulates transcripts of the *Drosophila* actin-binding protein, profilin (Chickadee, Chic) [104], which promotes the formation of F-actin [105]. The loss of *chic* enhances the *hep* (*Drosophila* JNKK) mutant dorsal open phenotype [104], and ectopic actin polymerization occurs in both *hep* and *chic* mutants suggesting that these may be acting via a common pathway to regulate cytoskeletal rearrangements [104]. Moreover, a positive feedback loop has been revealed to exist between JNK signalling and actin cytoskeletal regulators [89]. Furthermore, a link between JNK and integrins, which can promote actin assembly [106], has been described: JNK promotes expression of βPS integrin (encoded by *myospheroid*, *mys*) and αPS3 integrin (encoded by *scab*, *scb*), and the loss of *mys* and *scb* results in a similar dorsal open phenotype due to loss of JNK [107]. Therefore, in addition to the established function of Src in direct regulation of actin dynamics, additional signalling via the JNK pathway may also contribute to regulation of cell shape or cell adhesion via its effects on actin regulation. Additionally, F-actin reorganization can promote cell proliferation through inhibiting the Hippo pathway [93,108,109,110,111,112,113]. Furthermore, whilst JNK activity promotes cell death and activates the Hippo pathway, in the presence of Ras signalling, JNK signalling instead leads to Hippo pathway impairment via increased actin polymerization [114]. Src64B can also affect Hippo pathway signalling more directly through the actin cytoskeletal regulators, Rac1 and Diaphanous, which together with Src64B-induced Ras-MAPK signalling drives actin polymerisation, and when JNK signalling is impaired promotes tissue overgrowth in eye disc clones [44].

We show here that, in *Src + Ras^ACT^* clones, the activation of JNK promotes overall tissue growth, inhibition of differentiation and migratory-like phenotypes. In *Src + Ras^ACT^* cooperation, Ras most likely functions to suppress JNK-mediated cell death by inhibiting the apoptosis inducer, Hid [66,67,115], thereby revealing other functions of JNK signalling, such as the promotion of cell migration and inhibition of differentiation. Src expression in the embryo induces cell migration [46], and in corroboration with the potential role of JNK in migratory-like phenotypes in *Src + Ras^ACT^* tumours, the loss of JNK was shown to suppress the migratory effects arising from Src activation [116].

The finding that JNK is a critical component in the cooperation between *Src + Ras^ACT^*, correlates with previous analysis of *Drosophila* cooperative tumourigenesis with cell polarity or actin cytoskeletal regulators [51,71,72,74,90,91,92,93,94,100]. JNK is activated in *Ras^ACT^ + scrib*, *dlg* or *lgl* mutant tumours, and also promotes tissue growth and invasive phenotypes. Expression profiling has revealed a large number of JNK targets that affect cell differentiation in *Ras^ACT^ + scrib* mutant eye-antennal epithelial tissue [92,94,95,96], or in *scrib* homozygous wing epithelial tissue [117]. Moreover, JNK and Yorkie (Yki, a co-transcription factor inhibited by the Hippo pathway) mediated-upregulation of secreted factor dILP8 (*Drosophila* Insulin-Like Peptide 8), which inhibits the Ecdysone steroid hormone production from the prothoracic gland, results in the delayed larval-pupal transition caused by imaginal disc neoplastic tumours [94,118,119,120]. It is likely that similar mechanisms are induced in *Src* + *Ras^ACT^* tumours to result in differentiation defects and the developmental delay at the larval stage. In mammalian systems, a role for JNK in inhibition of differentiation has been reported [121,122]. JNK also plays a role in cell transformation induced by coexpression of *c-myc* (cellular-myc proto-oncogene) and *Ras^V12^* in mouse embryonic fibroblasts [123], although, these studies were carried out in vitro, and the precise effect of JNK signalling in *c-myc* and *Ras^V12^*-mediated oncogenic cooperation is unclear. However, based on the findings in *Drosophila* tumour models described here and previously [71,72,90,100], inhibiting JNK signalling may restore differentiation and suppress the malignant overgrowth and invasive characteristics in many human tumours. Indeed, in bRAF (b-RAF proto-oncogene)-driven melanomas, JNK-cJun (cellular-Jun proto-oncogene) signalling has been revealed to contribute to tumour progression, suggesting that blocking JNK signalling may be of therapeutic benefit in at least some cancer types [124,125,126]. However, although JNK clearly plays an important role in *Src + Ras^ACT^* tumourigenesis, the activation of JNK signalling with *Ras^ACT^* does not result in as aggressive tumours as with *Src* + *Ras^ACT^* [51], which might be due to the contribution of Src signalling to Hippo pathway impairment [44] or to possible effects of Src on the activation of Myosin II activity and actinomyosin cell contractility, cell shape changes and tissue growth [74].

Our discovery that *Raf^GOF^* was not sufficient to phenocopy *Ras^ACT^* in cooperation with *Src* was surprising, given that Raf expression is able to phenocopy Ras in cooperation with *scrib* mutant [72,100] and *RhoGEF2* [74]. Interestingly, we found that in the context of *Src* and *Ras^ACT^* cooperative tumourigenesis, the PI3K pathway is likely to play a critical role alongside contribution from Raf-MAPK signalling. Indeed, *dlg Ras^ACT^* tumours have compromised PI3K signalling and knockdown of PI3K pathway signalling is synthetically lethal to tumourigenesis [127]. Therefore, although *scrib Ras^ACT^* and *RhoGEF2 Ras^ACT^* tumours do not depend on Ras-driven PI3K signalling, it is possible that they are still sensitive to its depletion. The reason *Src* + *Ras^ACT^* tumourigenesis is dependent on PI3K signalling remains to be determined. One possible mechanism might relate to the importance of PI3K-mTOR (mechanistic Target of Rapamycin) signalling in blocking autophagy, a catabolic pathway that leads to the degradation of cellular components to produce energy [128]. Recently, polarity-impaired Ras-driven cancers have been shown to be dependent on induction of autophagy in neighbouring wild-type cells [129], which suggests that the PI3K-mTOR pathway might be important in this non-cell autonomous mechanism in tumour development. Additionally, the PI3K-mTOR-S6K pathway has been revealed to be critical in modulating metabolism from oxidative phosphorylation to aerobic glycolysis, which is important for neoplastic tumour progression [130]. Further studies will be required to determine if PI3K signalling may be important in regulating such biological processes in the *Src* + *Ras^ACT^* or other cooperative tumour models. Altogether, our work along with these other studies revealing the requirement of PI3K signalling in tumourigenesis in *Drosophila* models, suggests that targeting PI3K signalling might provide a novel therapeutic approach for *Src*-overexpressing or polarity-impaired *Ras*-driven cancers.

Although the Src proto-oncogene is associated with cancer [131], its precise role in tumour development and the significance of the contributions of its many downstream effectors to tumourigenesis remains unclear. Furthermore, given the strong correlation of elevated Ras protein expression in human tumours [132], examining the mechanism of cooperation between these key oncogenes may allow more precise targeting of critical signalling components, such as the JNK, Raf-MAPK and PI3K pathways, for improved therapies and better patient outcomes. Therefore, this *Drosophila* clonal model system has provided a robust in vivo setting in which to investigate Src function in cooperation with Ras^ACT^, and potentially could be utilized to gain further insight to other cooperative interactions identified in human disease.

## 4. Materials and Methods

### 4.1. Drosophila Stocks

Transgenes were overexpressed using *ey-GAL4* [56], *GMR-GAL4* [133] and *hsp70-GAL4* [134]. The *MARCM* system [70,135] was used to generate mosaic eye tissue using *w ey-FLP1*, *UAS-mCD8-GFP*; *tub-GAL4 FRT82B tub-GAL80*. Transgenic fly stocks employed were: *Src42A^GS^* [136], *UAS-Src42A* [50], *UAS-Src42A^ACT^* [50], *UAS-Src64B* (R. Cagan), *Src42A^EY08937^* [52], *UAS-Ras^ACT^* [61], *UAS-Raf^GOF^* [99], *UAS-bsk^DN^* [87], *UAS-p35* [68], *UAS-PTEN* [137] and *UAS-Dp110^DN^* [138]. *ey-GAL4* analysis using *p35* and *Ras^ACT^* were carried out using recombinants generated on the second chromosome carrying *ey-GAL4* and *UAS-p35* or *ey-GAL4* and *UAS-Ras^ACT^*.

### 4.2. Immunohistochemistry

Third instar larval eye imaginal discs were dissected and fixed in 6% (*w*/*v*) paraformaldehyde/HEPES for 20 min. Antibodies used were mouse anti-Elav (Developmental Studies Hybridoma Bank, 1:5), mouse anti-BrdU (Becton-Dickinson, Franklin Lakes, NJ, USA, 1:50) and rabbit anti-β-galactosidase (Rockland, 1:400), anti-*E*-cadherin (DSHB, 1:50), anti-aPKC-ζ (Upstate Biotechnology Inc., Lake Placid, NY, USA, 1:1000), anti-Discs Large (Dlg) (DSHB, 1:20), anti-Bazooka (Baz) [139], 1:200) and anti-GFP (Invitrogen, Carlsbad, CA, USA, 1:1000). F-actin was detected with phalloidin-tetramethylrhodamine isothiocyanate (TRITC; Sigma, St. Louis, MO, USA, 0.3 mM). For detection of apoptotic cells, a TUNEL assay (TMR, Roche, Basel, Switzerland) was conducted on eye imaginal discs that were permeabilized overnight at 4 °C in 0.3% (*v*/*v*) Triton X-100 in PBS according to manufacturer’s instructions. For the detection of S phase cells, a 1 h BrdU pulse at 25 °C was followed by 1 h fixation, immunodetection using anti-GFP antibody, further fixation, acid treatment using 0.1 M HCl, followed by detection of the BrdU epitope. All fluorescent labelled samples were captured by confocal microscopy (BioRad MRC 1000; Kidlington, Oxford, UK)) using Lasersharp 2000 software (Micro-Scientific, Gurnee, IL USA). Data were processed using Confocal Assistant (Purdue University Cytometry Laboratories, IN, USA) and Adobe Photoshop CS2 software (Adobe Systems, San Jose, CA, USA).

### 4.3. Adult Eye Imaging

Adult eyes were viewed under a light dissecting microscope and images captured on an Olympus DP11 camera, and data were processed using Adobe Photoshop CS2. For scanning electron microscope images, Adult flies were placed in a screw-cap tube in 25% (*v*/*v*) acetone for 1 h nutation at room temperature, followed by 50% (*v*/*v*) acetone for 2–3 h, 75% (*v*/*v*) acetone for a further 3 h and then finally placed in 100% acetone. Adult flies were critical point dried on a Balters CPD 030 Critical Point Dryer and coated with gold particles in an Edwards 6150B Gold Sputter Coater (kindly carried out by Simon Crawford, Department of Botany, University of Melbourne). Images were recorded on a Phillips XL30 FEG Field Emission Electron Microscope (Amsterdam, Netherlands).

### 4.4. Western Analysis

Protein expression was induced in third instar larvae by 1 h heat shock at 37 °C where *hsp70-GAL4* was used to drive expression of *UAS-Src42A*, *UAS-Src42A^GS^* or no transgene control (*w^1118^*). After 1 h recovery at 25 °C, larval eye tissue was dissected and lysed in NTEN buffer with fresh protease inhibitor. Protein lysates were subjected to SDS-PAGE and immunoblotted with anti-phosphorylated Src (against the autophosphorylated Tyrosine residue in the kinase domain, indicating active Src (anti-pSrc, Biosource, 1:1000)), anti-*Drosophila* Src42A to detect expression levels (α-Src42A, [58], 1:1000), and anti-tubulin to indicate protein loading (anti-tubulin, Calbiochem, 1:10,000). Protein bands on Western blots were quantified using Image J (National Institutes of Health, Bethesda, MA, USA).

## Figures and Tables

**Table 1:** Summary of adult eye phenotypes produced by Src transgene expression using *ey-GAL4.*

	Transgene	Control	*Src42A^GS^*	*Src42A*	*Src42A^ACT^*	*Src64B*
*GAL4* driver						
*ey>*		Wild-type	Wild-type	Wild-type	Range of rough eye phenotypes, from very small to reduced eye size	Range of rough eye phenotypes, from no eye to smaller reduced size
*ey > p35*		Wild-type	Wild-type	Wild-type	Rough, reduced eye size	Rough, reduced eye size
*ey > Ras^ACT^*		Hyperplastic overgrowth	Enhanced hyperplastic overgrowth	No enhanced overgrowth	Enhanced growth in dorsal region, reduced in ventral region	Enhanced hyperplastic overgrowth

**Table 2:** Summary of phenotypes of *ey-FLP MARCM* third instar eye clones expressing *Src42A^GS^* with the indicated transgenes.

	Transgene	*Src42A^GS^* +				
Phenotype		Control	*p35*	*bsk^DN^*	*Ras^ACT^*	*Ras^ACT^ bsk^DN^*
Clone size		Small clones; increased cell death	Small clones, with increased cell proliferation in adjacent wild-type clones	Increased clone size, and basal overgrowth	Large clones, wild type tissue present	Reduced clonal overgrowth; restored tissue morphology
Differentiation		Normal	Normal but disrupted organisation	Normal but disrupted organisation	Reduced	Restored
F actin		Normal, some clones show enriched F-actin	Accumulation	Cortical localisation	Accumulation	Cortical localisation
Protrusive morphology		Yes	NA	NA	Enhanced	Suppressed
JNK (Jun N-terminal kinase) pathway reporter		NA	Increased	NA	Increased	NA
Adult phenotype		1–2 day delay in adult eclosion, eye phenotype comparable to control	Overgrown eye tissue	Lethal at late larval/early pupal stage	Lethal at late L3, with melanotic masses	Lethal at late larval/early pupal stage

NA = not assessed.

**Table 3:** Summary of phenotypes of *ey-FLP MARCM* third instar eye clones expressing *Src64B* with the indicated transgenes.

	Transgene	*Src64B* +				
Phenotype		Control	*p35*	*bsk^DN^*	*Ras^ACT^*	*Ras^ACT^ + bsk^DN^*
Clone size		Small clones within epithelium; Discrete, rounded clones excluded from epithelia proper; increased cell death	Small clones, clones, with increased cell proliferation in adjacent wild-type clones	Increased clone size, basal over-growth	Large clones that out compete wild -type tissue	Reduced clone size; restored overall tissue morphology
Differentiation		Normal differentiation in small clones; no differentiation in clones located apical to eye disc proper	Normal but disrupted organisation	Normal but disrupted organisation, with some differentiated cells localised basally	Reduced	Partially restored
F-actin		Accumulation	Accumulation	Reduced cortically	Accumulation	Enriched cortically
Protrusive morphology		NA	NA	NA	Increased	Decreased
JNK pathway reporter		Increased	NA	NA	Increased	NA
Adult phenotype		1–2 day delay in adult eclosion, eye phenotype comparable to control	Larval lethal	Lethal at late larval/early pupal stage, with melanotic masses	Larval lethal	Lethal at late larval/early pupal stage

NA = not assessed.

**Table 4:** Summary of phenotypes of *ey-FLP MARCM* third instar eye clones expressing *Src64B* with indicated transgenes.

	Transgene	*Src64B* +			
Phenotype		*Raf^GOF^*	*Ras^ACT S35^*	*Ras^ACT^ + PTEN*	*Ras^ACT^ + Dp110^DN^*
Clone size		No cooperative overgrowth	Large clones	Reduced clone size compared to *Src + Ras^ACT^*	Reduced clone size compared to *Src + Ras^ACT^*
Differentiation		Suppressed ectopic *Raf^GOF^* differentiation	Reduced	Reduced	Reduced
F-actin		Enrichment at clone borders	Accumulation	Accumulation	Accumulation

## Supplementary Materials

Supplementary materials can be found at http://www.mdpi.com/1422-0067/19/6/1585/s1.

## Abbreviations

Baz	Bazooka
Csk	C-terminal Src-like kinase
Dlg	Discs-large
EGF	Epidermal growth factor
EGFR	Epidermal growth factor receptor
JNK	Jun N-terminal Kinase
MMP	Metalloproteinase
MAPK	Mitogen-activated protein kinase
PI3K	PhosphoInoitide 3-kinase
