# Supplementary material for: Src Cooperates with Oncogenic Ras in Tumourigenesis via the JNK and PI3K Pathways in Drosophila epithelial Tissue
Source: Int J Mol Sci. 2018 May 27;19(6):1585. doi: 10.3390/ijms19061585 (PMC6032059; doi:10.3390/ijms19061585)
Supplement: Supplementary file 1 [file ijms-19-01585-s001.pdf]

Supplementary Figure Legends

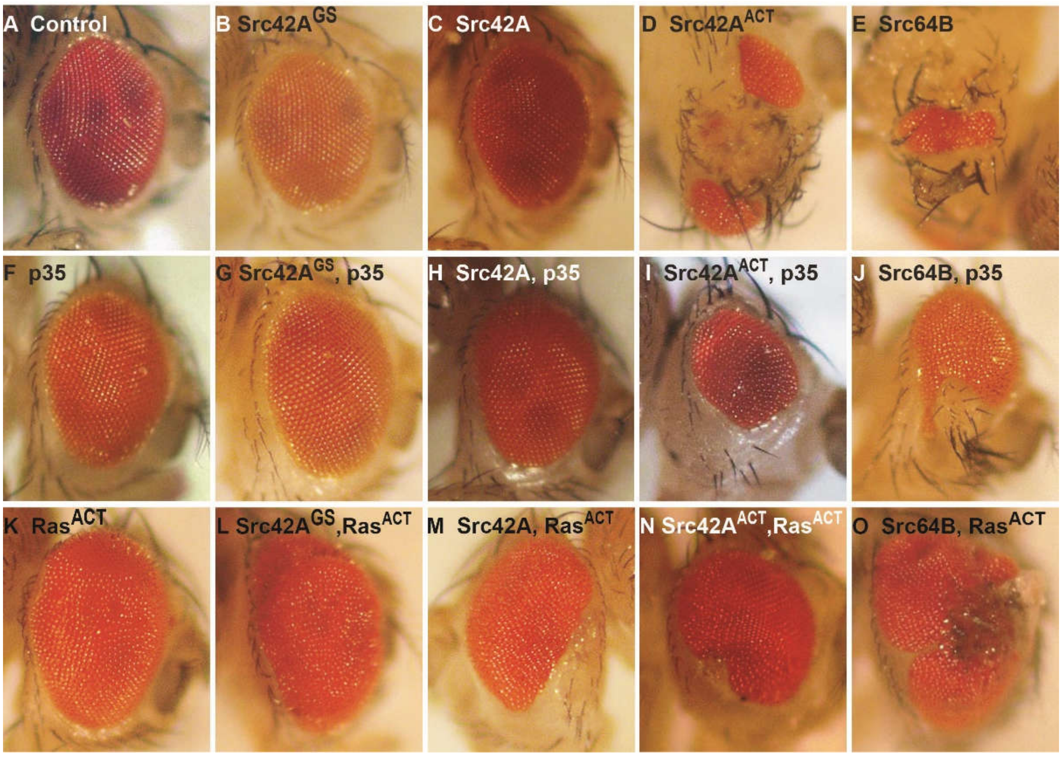

Figure S1. Cooperation between *Src* + *Ras*^{ACT} in the *ey-GAL4* system requires more than cell survival signals from *Ras*^{ACT}. Light micrographs of male adult eyes in lateral view bearing the genotypes: (A) Control: *ey-GAL4*/+, (B) Src42A^{GS}: *ey-GAL4*/ *GS11049*, (C) Src42A: *ey-GAL4*/ +; *UAS-Src42A*/ +, (D) Src42A^{ACT}: *ey-GAL4*/ +; *UAS-Src42A*^{ACT} / +, (E) Src64B: *ey-GAL4*/ *UAS-Src64B*, (F) p35: *ey-GAL4,UAS-p35*/ +, (G) Src42A^{ACT}, p35: *ey-GAL4, UAS-p35*/ *GS11049*, (H) Src42A, p35: *ey-GAL4, UAS-p35*/ +; *UAS-Src42A*/ +, (I) Src42A^{ACT}, p35: *ey-GAL4, UAS-p35*/ +; *UAS-Src42A*^{ACT}/ +, and (J) Src64B, p35: *ey-GAL4, UAS-p35*/ *UAS-Src64B*, (K) Ras^{ACT}: *ey-GAL4, UAS-Ras*^{ACT} /+, (L) Src42A^{GS}, Ras^{ACT}: *ey-GAL4, UAS-Ras*^{ACT}/ *GS11049*; (M) Src42A, Ras^{ACT}: *ey-GAL4*, *UAS-Ras*^{ACT} /+; *UAS-Src42A* /+, (N) Src42A^{ACT}, Ras^{ACT}: *ey-GAL4*, *UAS-Ras*^{ACT} /+; *UAS-Src42A*^{ACT}/+ and (O) Src64B, Ras^{ACT}: *ey-GAL4*, *UAS-Ras*^{ACT} /+; *UAS-Src64B*/ +. Expression of *Src42A*^{GS} (B) or *Src42A* (C) using *ey-GAL4* did not discernibly affect the adult eye phenotype compared to the control (A), whereas expression of *Src42A*^{ACT} (D) or *Src64B* (E) resulted in a reduced adult eye size. Expression of caspase inhibitor *p35* (F) using *ey-GAL4* resulted in adult eyes comparable to the control (A). There was no discernible difference when *p35* was coexpressed with *Src42A*^{GS} (G) or *Src42A* (H) compared to expression of *Src42A*^{GS} (B) or *Src42A* (C) alone, respectively. However, there was partial suppression when *p35* was coexpressed with *Src42A*^{ACT} (I) or *Src64B* (J). Expression of *Ras*^{ACT} (K) resulted in hyperplastic adult eyes compared with the control (A). *ey*-driven coexpression of *Src42A*^{GS} + *Ras*^{ACT} (L) enhanced the *Ras*^{ACT} hyperplastic eye phenotype resulting in enlarged, folded eyes. Coexpression of *Src42A* + *Ras*^{ACT} (M) did not enhance the *Ras*^{ACT} hyperplastic eye phenotype (K), whereas coexpression of *Src42A*^{ACT} with *Ras*^{ACT} (N) resulted in weak enhancement of the hyperplastic *Ras*^{ACT} eye phenotype, predominantly in the dorsal region. Expression of *Src64B* also enhanced the *ey-GAL4,UAS-Ras*^{ACT} hyperplastic eye phenotype (O) resulting in an enlarged adult eye characterised by enhanced overgrowth and aberrant cuticle.

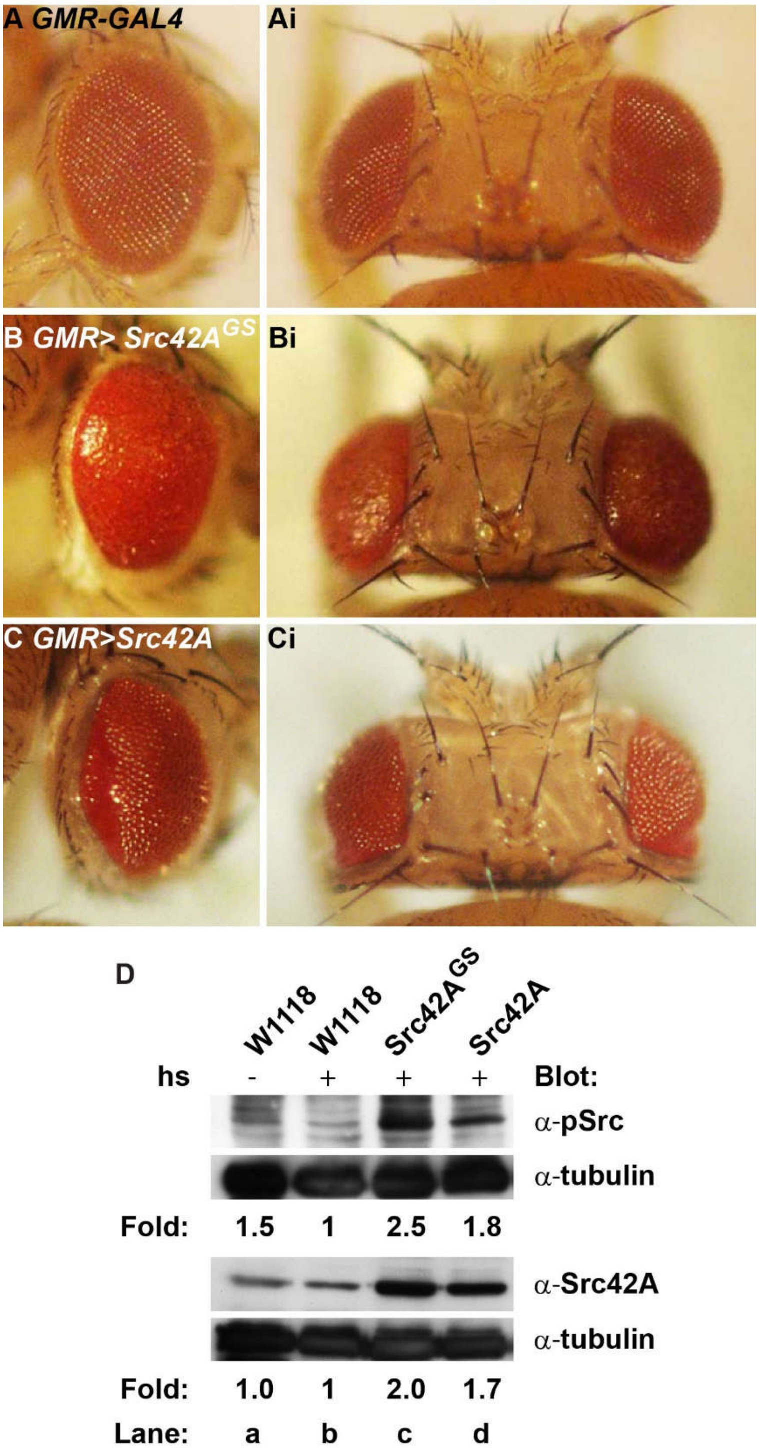

Figure S2. *GMR*-driven expression of $Src42A^{GS}$ resulted in stronger induction of Src protein and activity compared to expression of the *Src42A* transgene. A-C: Light micrographs of male adult eyes bearing the genotypes: (A) *GMR-GAL4* control: *GMR-GAL4*/+, (B) *GMR-GAL4*, $Src42A^{GS}$: *GMR-GAL4*/ *GS11049*, (C) *GMR-GAL4*, Src42A: *GMR-GAL4*/ +; *UAS-Src42A*/ +. Lateral view (first column) and dorsal view (i, second column). Expression of $Src42A^{GS}$ with *GMR-GAL4* resulted in a glazed, glassy adult eye (B) that was slightly overgrown compared with the *GMR-GAL4* driver alone (A). Expression of an independent transgene of *Src42A* with *GMR-GAL4* resulted in a slightly smaller adult eye (C), with a small, glazed stripe in the posterior region of the eye. D: Western analysis: Protein expression was induced at third instar larval stage after 1 hr heat shock, and lysates collected after 1 hr recovery. 20µg of protein was analysed by Western blotting with the following antibodies: anti-phosphorylated Src (against the autophosphorylated Tyrosine residue in the kinase domain, indicating active Src (α-pSrc), anti-*Drosophila* Src42A to detect expression levels (α-Src42A), and anti-tubulin to indicate

protein loading (α-tubulin). Control lanes (lanes a and b) indicated low basal Src activity (α-pSrc) and moderate levels of endogenous Src42A protein (α-Src42A). Heat shock induction of *Src42A^{GS}* (lane c) and *Src42A* (lane d) resulted in increased pSrc levels by 2.5 and 1.8 fold, respectively, and increased protein expression by 2.0 and 1.7 fold, respectively, compared to control lane b. Comparsion between the two wild-type *Src42A* lines showed that *Src42A^{GS}* (lane c) resulted in a higher level of autophosphorylation (α-pSrc) and expression (α-Src42A) compared with Src42A (lane d). To quantitate fold differences, densitometry was measured using ImageJ software. pSrc and Src42A were normalised to their respective tubulin controls; and fold differences were normalised to the heat-shocked negative control (lane b).

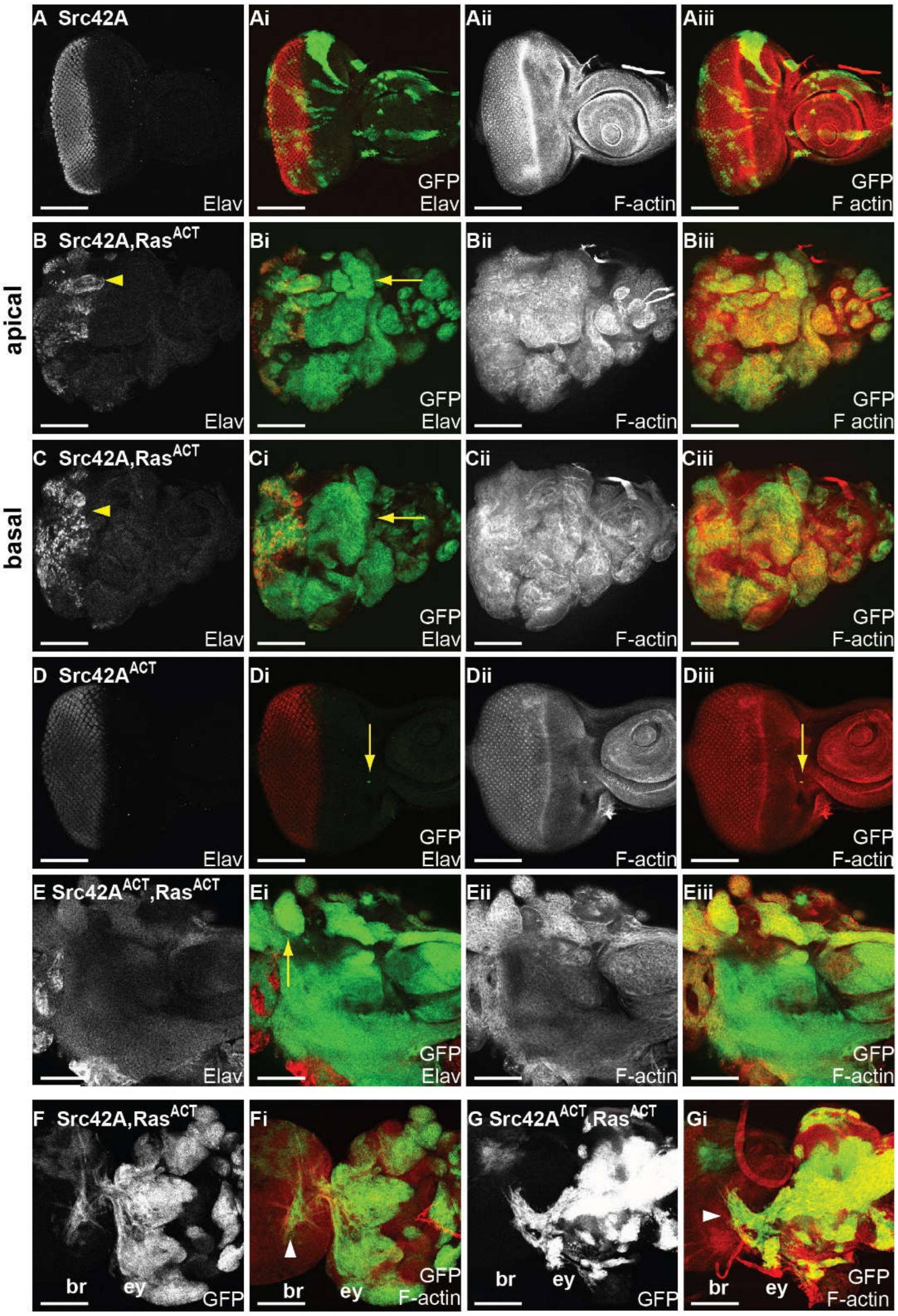

Figure S3. Expression of *Src42A* + *Ras^{ACT}* or *Src42A^{ACT}* + *Ras^{ACT}* in eye disc clones results in cooperative overgrowth. Planar confocal images of eye-antennal discs. Clones are marked by expression of GFP (green in merged image). A-E: Elav marks the nuclei of developing photoreceptors (red in second column), rhodamine-phalloidin marks F-actin (A-E: red in fourth column). F-G: br – brain lobe, ey –

eye antennal disc, rhodamine-phalloidin is red in merged image. 20x magnification, 50μM scale bar. Genotypes: (A) Src42A: *ey-FLP1, UAS-mCD8-GFP/ +; ; tub-GAL4 FRT82B tub-GAL80/ FRT82B UAS-Src42A*, (B, C, F) Src42A, Ras^{ACT}: *ey-FLP1, UAS-mCD8-GFP/ +; UAS-Ras*^{ACT}*/ +; tub-GAL4 FRT82B tub-GAL80/ FRT82B UAS-Src42A*, (D) Src42A^{ACT}: *ey-FLP1, UAS-mCD8-GFP/ +; ; tub-GAL4 FRT82B tub-GAL80/ FRT82B UAS-Src42A*^{ACT}, (E, G) Src42A^{ACT}, Ras^{ACT}: *ey-FLP1, UAS-mCD8-GFP/ +; UAS-Ras*^{ACT}*/ +; tub-GAL4 FRT82B tub-GAL80/ FRT82B UAS-Src42A*^{ACT}. B is an apical section and C is a basal section of one sample of *Src42A* + *Ras*^{ACT}. Expression of *Src42A* (A) resulted in mosaic eye discs generally comparable to control mosaic eye discs (Figure 2A-B) with relatively normal differentiation (Ai) and F-actin organisation (Aii-iii). Expression of *Src42A*^{ACT} reduced clonal size (yellow arrow, Di), which prevented analysis of the effects on differentiation (Di) or F actin organisation (Diii). Coexpression of *Src42A* + *Ras*^{ACT} (B-C) or *Src42A*^{ACT} + *Ras*^{ACT} (E) resulted in both increased clone size and overall eye tissue overgrowth. Rounded clones were observed (yellow arrow, B-C) and larger masses of tissue localised in basal regions of the epithelium (arrow, C and data not shown for *Src42A*^{ACT} + *Ras*^{ACT}). There was a general loss of differentiation in eye discs expressing *Src42A* + *Ras*^{ACT} (B-Ci-ii) or *Src42A*^{ACT} + *Ras*^{ACT} (Ei-ii). Some differentiation occurred in both wild-type and clonal tissue, although the patterning was severely disrupted and the morphogenetic furrow was less discernible. The normally apical differentiation marker Elav was observed in apical and basal sections (yellow arrowhead, A-Bi) of both wild-type and clonal tissue coexpressing *Src42A* + *Ras*^{ACT}. Expression of *Src42A* + *Ras*^{ACT} (B-Ciii-iv) or *Src42A*^{ACT} + *Ras*^{ACT} (Eiii-iv) resulted in disruption to F-actin organisation. Clonal tissue showed increased F-actin staining (B-Cii-iii, Eii-iii). Extended projections were observed in *Src42A* + *Ras*^{ACT} (F) or *Src42A*^{ACT} + *Ras*^{ACT} (G) clonal tissue in the brain lobe (br) and were enriched for F-actin (arrowhead, F-Gi).

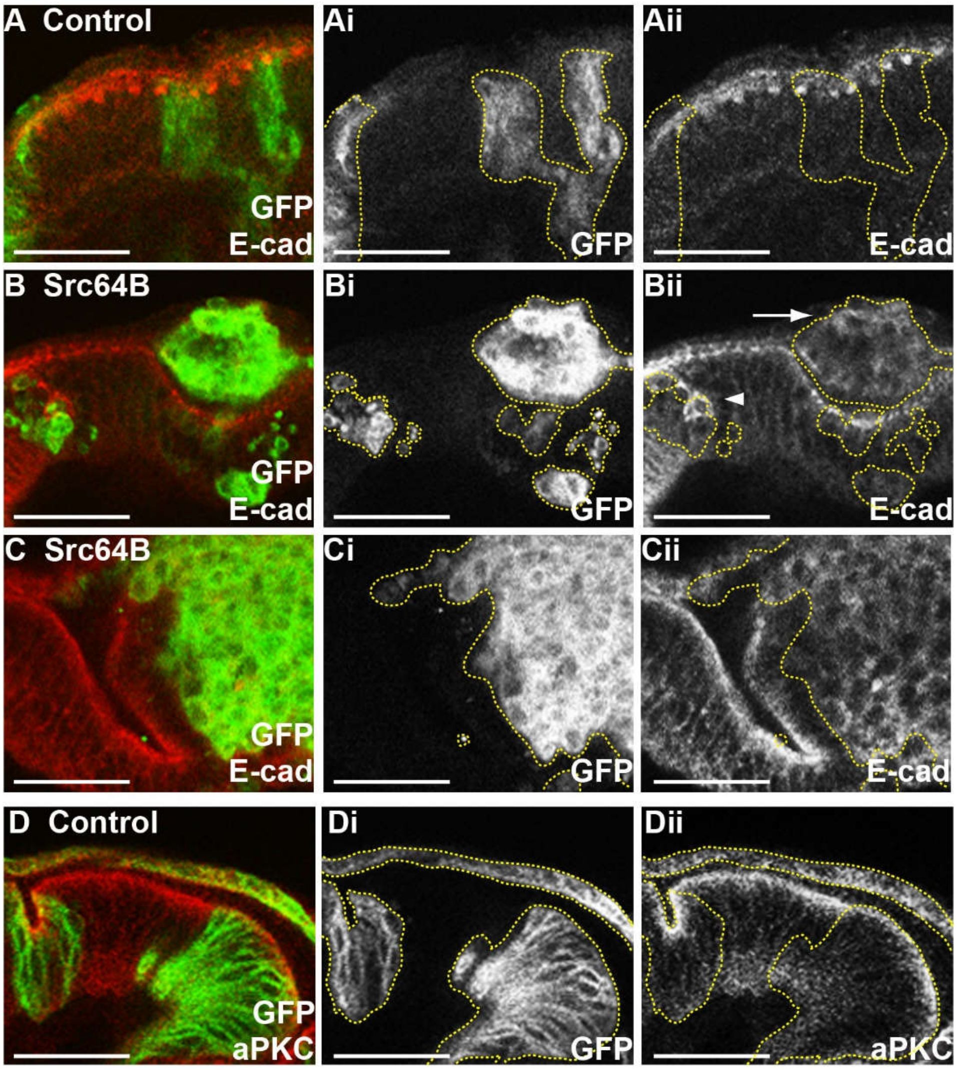

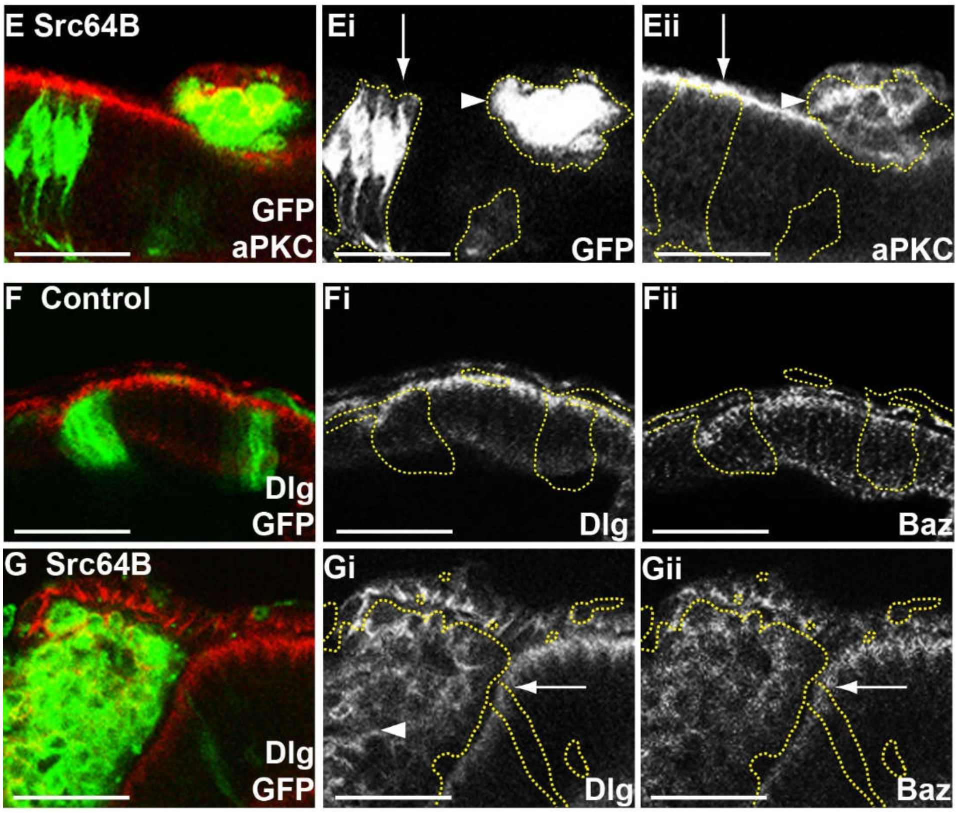

Figure S4. Expression of *Src64B* in eye disc clones results in mislocalised cell polarity determinants**.** Confocal images, cross sections, prepared from side-mounted eye discs, oriented with apical surface to the top, and basal to the bottom. Clones are marked by expression of GFP (green in merged image, and outlined in yellow dashed line). A-C: E-cadherin (E-cad) marks adherens junctions (A-Cii, red in merged images), D-E: aPKC localises to the subapical region (D-Eii, red in merged images), F-G: Discs large (Dlg) marks septate junctions (F-Gi, red in merged images) and Bazooka (Baz) is a subapical marker (F-Gii). 40x magnification, 25 μM scale bar. Genotypes: (A, D, F) Control: *ey-FLP1, UAS-mCD8-GFP/* +; +; *tub-GAL4 FRT82B tub-GAL80/ FRT82B*, (B, C, D, G) Src64B: *ey-FLP1, UAS-mCD8-GFP/* +; *UAS-Src64B/* +; *tub-GAL4 FRT82B tub-GAL80/ FRT82B*. E-cadherin marked adherens junctions in wild-type cross sections (A). Expression of *Src64B* resulted in diffuse and mislocalised E-cadherin; larger clones showed some E-cadherin around the cell surface but this was not always uniform (white arrow, B-C, ii). A smaller clone within the epithelia is outlined with E-cadherin (white arrowhead, Bii). In control mosaic discs, aPKC localised to the subapical region of the epithelium (D). In *Src64B* mosaic eye discs, diffuse aPKC staining was observed in cells within larger clones (white arrowhead, Ei), whereas *Src64B* clonal tissue that was located within the epithelium proper showed subapical aPKC, comparable to that observed in control mosaic discs (white arrow, Ei-ii). In control mosaic eye discs, Dlg localised to the septate junction towards the apical surface of the epithelium (Fi), and Baz localised to the subapical region (Fii). The rounded *Src64B* clones showed a generally diffuse Dlg staining (Gi); in some cells, a distinct enrichment of Dlg was observed (arrowhead, Gi). Baz was generally diffuse in *Src64B* clones (Gii), but was correctly localised to the subapical region in surrounding wild-type tissue. When *Src64B* clones formed within the epithelium proper, both Dlg (arrow, Gi) and Baz (arrow, Gii) were correctly localised towards the apical surface as observed in their adjacent wild-type counterparts.

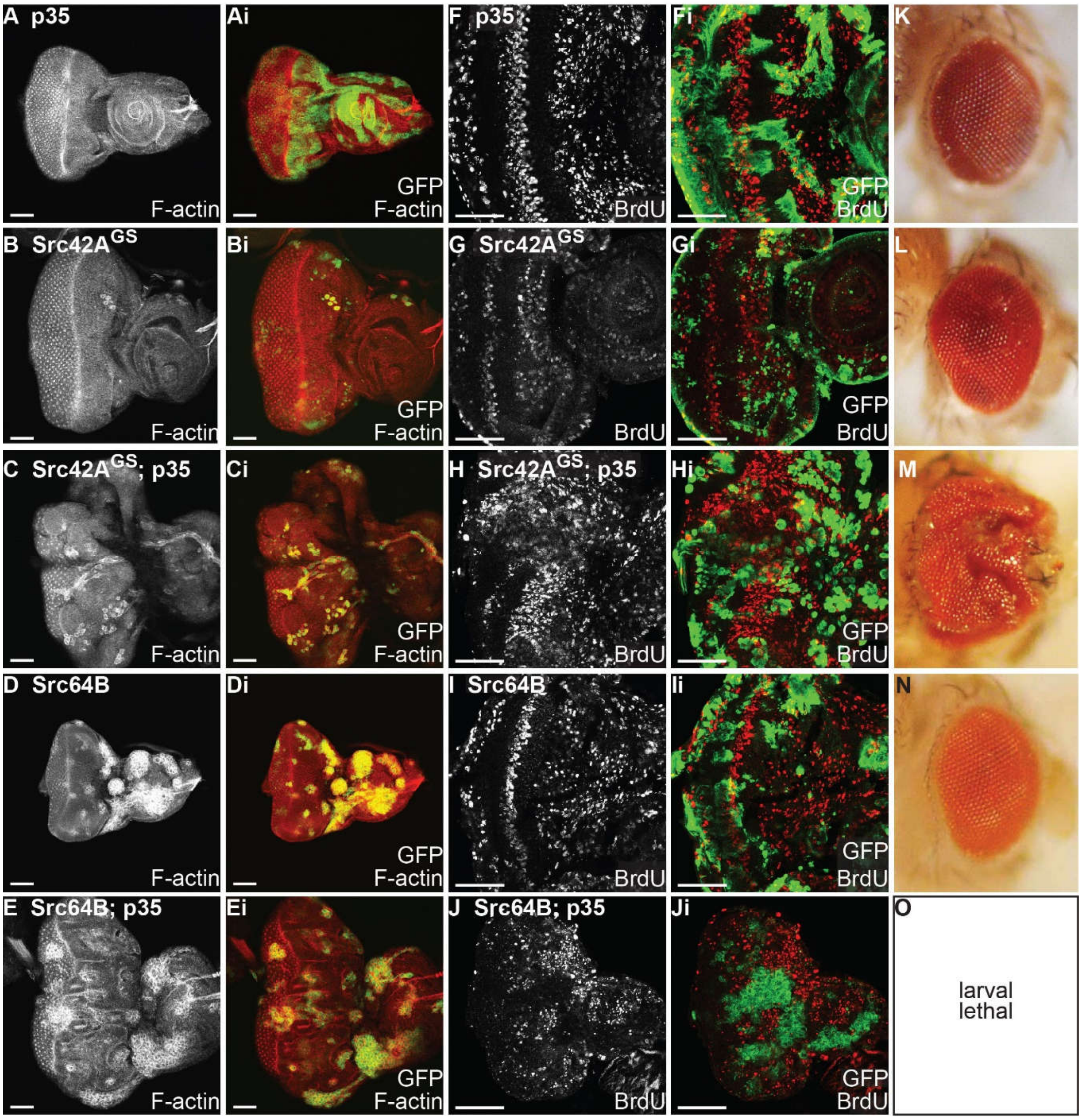

Figure S5. Expression of *Src* + *p35* in eye disc clones results in non-cell autonomous cell proliferation**.** Confocal planar images of eye-antennal discs. Clones are marked by expression of GFP (green in merged images). A-E: 20x magnification, 50μM scale bar. BrdU incorporation identifies proliferating cells (third column, red in merged image, i) and rhodamine-phalloidin visualises F-actin (first column, red in merged image, i). F-G: 40x magnification, 50μM scale bar. Proliferation was measured by BrdU incorporation assay to visualise cells undergoing S phase (left column, red in merged image). Light micrographs show lateral view of adult males, posterior to the left. Genotypes: (A, F, K) p35: *ey-FLP1, UAS-mCD8-GFP/ +; +; tub-GAL4 FRT82B tub-GAL80/ FRT82B UAS-p35*, (B, G, L) Src42A^{GS}: *ey-FLP1, UAS-mCD8-GFP/ +; GS11049/ +; tub-GAL4 FRT82B tub-GAL80/ FRT82B*, (C, H, M) Src42A^{GS}; p35: *ey-FLP1, UAS-mCD8-GFP/ +; GS11049/ +; tub-GAL4 FRT82B tub-GAL80/ FRT82B UAS-p35*, (D, I, N) Src64B: *ey-FLP1, UAS-mCD8-GFP/ +; UAS-Src64B/ +; tub-GAL4 FRT82B tub-GAL80/ FRT82B* and (E, J) Src64B; p35; *ey-FLP1, UAS-mCD8-GFP/ +; UAS-Src64B/ +; tub-GAL4 FRT82B tub-GAL80/ FRT82B UAS-p35.* **E**xpression of *p35* in clones did not discernibly alter F-actin organisation of the eye disc (A) compared to control mosaic eye discs (Figure 2B). Clonal size was marginally larger when *p35* was expressed with *Src42A^{GS}* (GFP, C) compared with expression of *Src42A^{GS}* alone but clonal size remained smaller than in control mosaic eye discs (Figure 2B). F-actin was increased and outlined clonal cells (Ci) and the surrounding wild-type tissue was overgrown and folded. As with expression of *Src64B* alone (D), cells expressing *Src64B* + *p35* were rounded and show an increase in F-actin (E). The overall morphology of the eye disc was altered leading tissue folding of the eye disc was reflected

in F-actin staining (Ei). F-actin also outlined the rounded cells within clonal tissue (Ei). Expression of *p35* (F) resulted in a similar pattern of proliferation (represented by S phase) to control mosaic eye discs (data not shown) with a band of S phase cells in the second mitotic wave and, posterior to the morphogenetic furrow, asynchronously cycling cells in the anterior. Expression of *Src42A*^{GS} (G) or *Src64B* (I) in eye disc clones did not discernibly affect S phase patterning compared to *p35* mosaic eye disc control (F), and resulted in normal adult eye phenotype (L and N, respectively). Coexpression of *Src42A*^{GS} + *p35* (H) or *Src64B* + *p35* (J) resulted in loss of the normal pattern of proliferation and an increase in S phases in the surrounding wild-type tissue rather than within clonal tissue. The resulting *Src42A*^{GS} + *p35* adult eye phenotype was folded and overgrown (M), reflecting the overproliferation observed in the wild-type tissue during the larval stage. Expression of *Src64B* + *p35* in clones resulted in lethality during third instar larval stage (O).

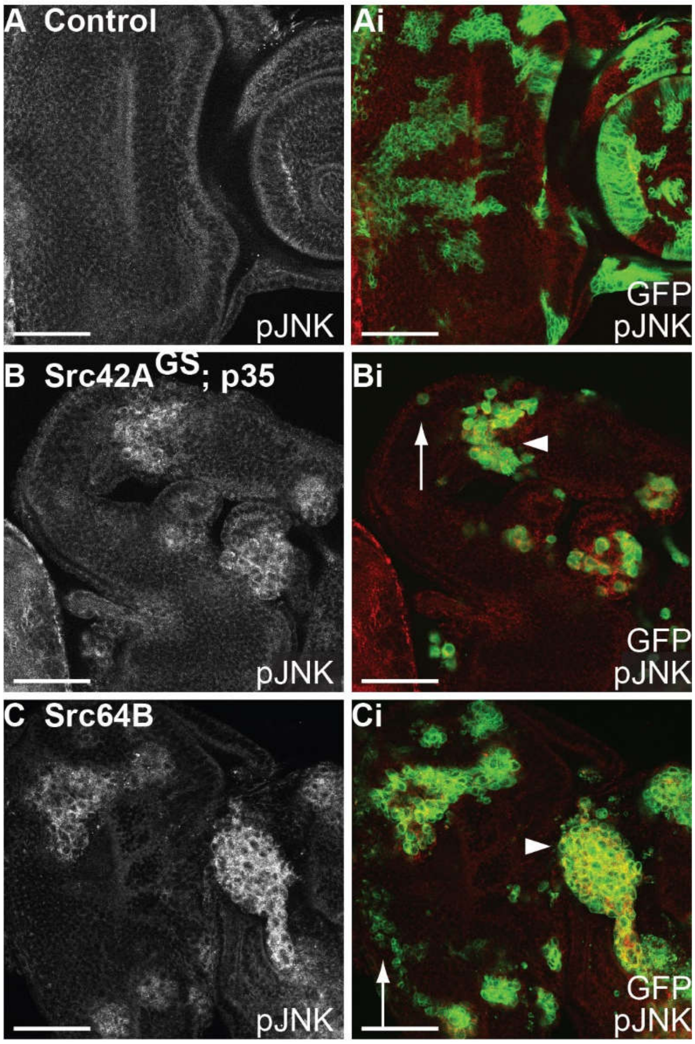

Figure S6. Expression of *Src42A* or *Src64B* activates JNK. Confocal images of planar views of eye-antennal discs. Clones are marked by expression of GFP (left column, green in merged images). Anti-phosphorylated-JNK (pJNK) antibody measures active JNK protein (red in merged images). 40x magnification, 50 µM scale bar. Genotypes: (A) Control: *ey-FLP1, UAS-mCD8-GFP*; +; *tub-GAL4 FRT82B tub-GAL80/ FRT82B*, (B) Src42A^{GS}; p35: *ey-FLP1, UAS-mCD8-GFP/* +; *GS11049/* +; *tub-GAL4 FRT82B tub-GAL80/ FRT82B UAS-p35*, (C) Src64B: *ey-FLP1, UAS-mCD8-GFP/* +; *UAS-Src64B/* +; *tub-GAL4 FRT82B tub-GAL80/ FRT82B*. pJNK signal in control mosaic eye imaginal discs was not significant (A). pJNK staining was increased within clonal tissue expressing *Src42A*^{GS} (arrowhead, Bi, with *p35* to increase clonal tissue size) or *Src64B* (arrowhead, Ci), in comparison with control mosaic eye antennal discs (A). pJNK signal was not discernible in small clones (arrows, Bi, Ci).

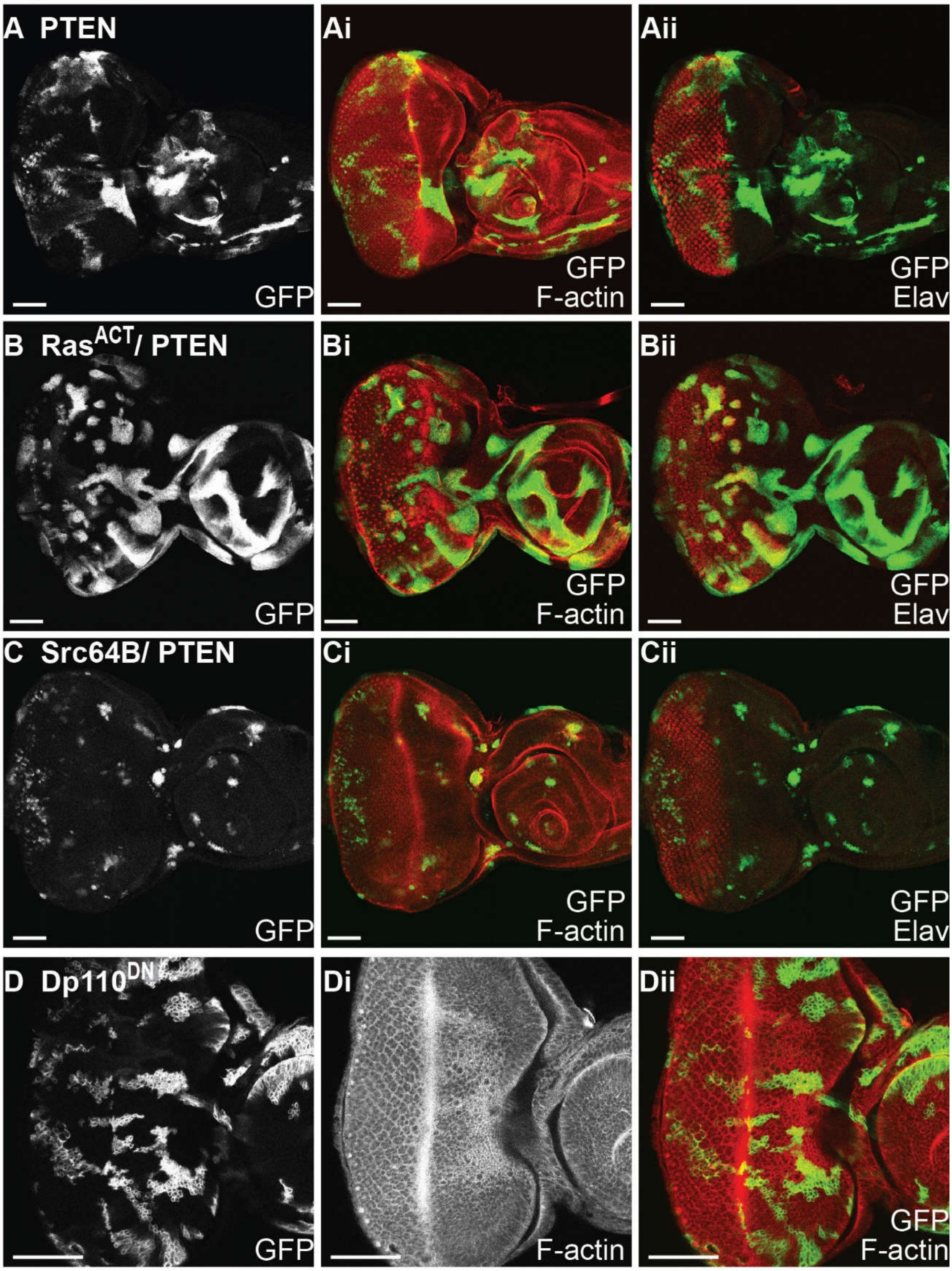
A PTEN
GFP
Ai
GFP
F-actin
Aii
GFP
Elav
B Ras^ACT/ PTEN
GFP
Bi
GFP
F-actin
Bii
GFP
Elav
C Src64B/ PTEN
GFP
Ci
GFP
F-actin
Cii
GFP
Elav
D Dp110^DN
GFP
Di
F-actin
Dii
GFP
F-actin

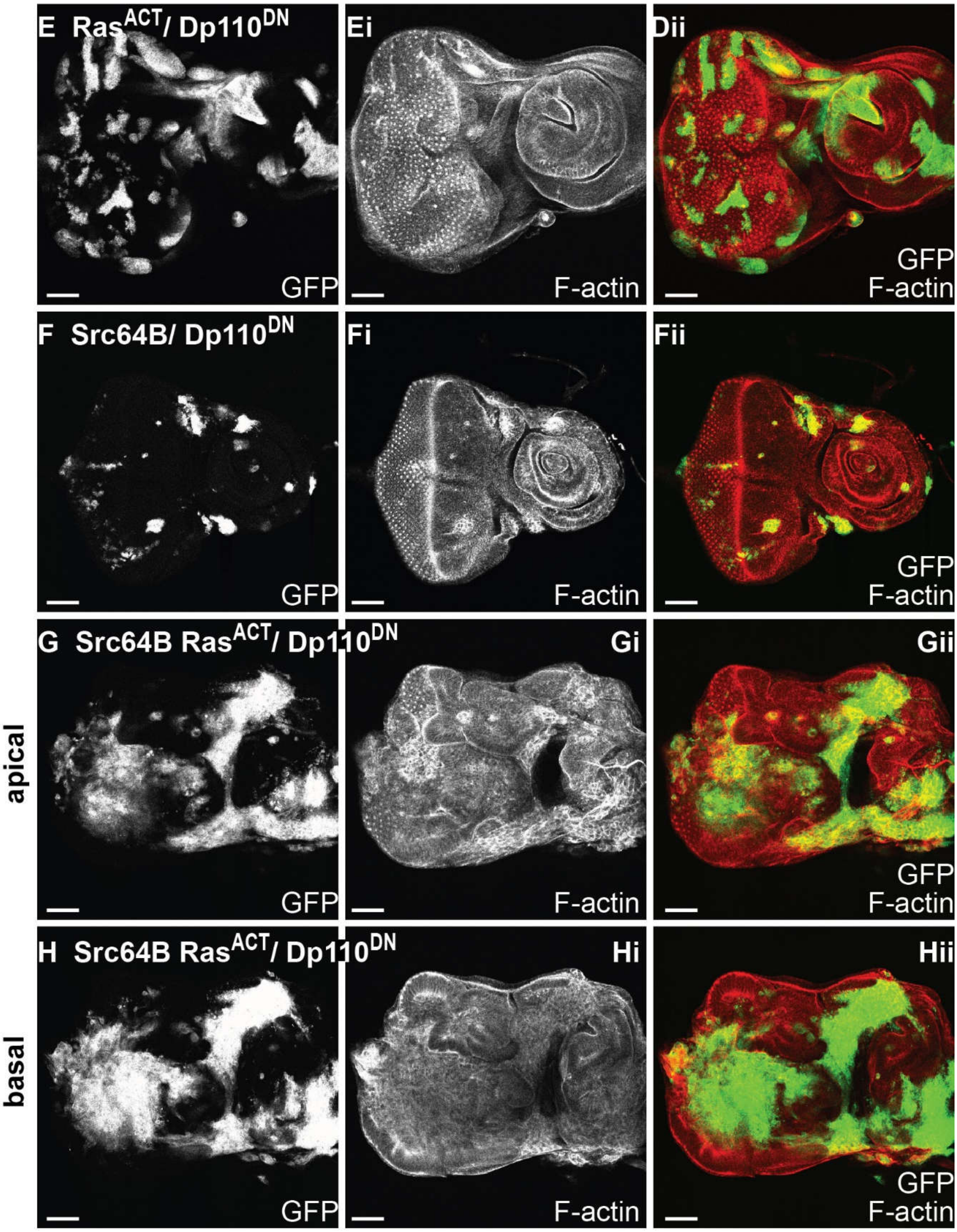

Figure S7. Blocking Dp110 signalling suppresses the cooperative overgrowth of *Src* + *Ras*^{ACT} in eye disc clones. Confocal images of planar views of eye-antennal discs. Clones are marked by expression of GFP (green in merged images). Elav marks differentiated cells (red in merged images, Aii-Cii) and rhodamine-phalloidin visualises F-actin to mark cell outlines (in A-H as marked, red in merged images, Ai-Ci, and Dii-Hii). G is an apical view, and H is a basal view. A-H: 20x magnification, 50μM scale bar, except D: 40x magnification, 50μM scale bar. Genotypes: (A) PTEN: *ey-FLP1, UAS-mCD8-GFP/+; UAS-PTEN/+; tub-GAL4 FRT82B tub-GAL80/ FRT82B*, (B) Ras^{ACT} / PTEN: *ey-FLP1, UAS-mCD8-GFP/ +; UAS-Ras*^{ACT}*/ UAS-PTEN; tub-GAL4 FRT82B tub-GAL80/ FRT82B*, (C) Src64B/ PTEN: *ey-FLP1, UAS-mCD8-GFP/ +; UAS-Src64B/ UAS-PTEN ; tub-GAL4 FRT82B tub-GAL80/ FRT82B*, (D) Dp110^{DN}: *ey-FLP1, UAS-mCD8-GFP/ +; UAS-Dp110*^{DN}*/ +; tub-GAL4 FRT82B tub-GAL80/ FRT82B*, (E) Ras^{ACT}/ Dp110^{DN}: *ey-FLP1, UAS-mCD8-GFP/ +; UAS-Ras*^{ACT}*/ UAS-Dp110*^{DN}*; tub-GAL4 FRT82B tub-GAL80/*

FRT82B, (F) Src64B/ Dp110^{DN}: *ey-FLP1, UAS-mCD8-GFP/ +; UAS-Src64B/ UAS-Dp110^{DN}; tub-GAL4 FRT82B tub-GAL80/ FRT82B* and (G, H) Src64B.Ras^{ACT} / Dp110^{DN}: *ey-FLP1, UAS-mCD8-GFP/ +; UAS-Src64B. UAS-Ras^{ACT}/ UAS-Dp110^{DN}; tub-GAL4 FRT82B tub-GAL80/ FRT82B.* Expression of *PTEN* results in reduced size of GFP-marked clones (A) compared with control mosaic eye discs (Figure 2A). Expression of *PTEN* with *Ras^{ACT}* results in rounded clones and ectopic differentiation (Bii) comparable to that observed in mosaic eye discs expressing *Ras^{ACT}* alone (Figure 2C). Expression of *Src64B* with *PTEN* results in reduced clonal tissue size compared to expression of *Src64B* alone in eye disc clones (Figure 2I-J, 4G). The differentiation pattern of *Src64B* + *PTEN* clonal tissue (Figure S7C) was comparable to control mosaic eye discs (Figure 2A). Expression of dominant negative *Dp110*, *Dp110^{DN}*, in eye disc clones does not discernibly affect clonal tissue size (GFP-marked) or F-actin organisation (Di-ii). Coexpression of *Dp110^{DN}* with *Ras^{ACT}* in mosaic eye discs results in rounded clones with disruption to F-actin organisation (Ei-ii) comparable to that observed in eye disc clones expressing *Ras^{ACT}* alone (Figure 2C-D). Expression of *Src64B* with *Dp110^{DN}* (F) results in reduced clonal tissue size compared with expression of *Src64B* alone (Figure 2I-J, 4G). However, Src64B/ *Dp110^{DN}* clones were still enriched F-actin in GFP-marked clones (Fii), similar to that observed for clones expressing *Src64B* alone (Figure 2J, 4Gii, Hii, I). Coexpression of *Dp110^{DN}* with *Src64B* + *Ras^{ACT}* in eye disc clones results in a decrease in GFP-marked clonal tissue (G-H) compared with expression of *Src64B* + *Ras^{ACT}* (Figure 2L). Rounded cells are outlined with F-actin in *Src64B* + *Ras^{ACT}* + *Dp110^{DN}* clonal tissue, and in the apical view, photoreceptors (marked by F-actin) are discernible in wild-type tissue in the posterior region of the eye disc, but not in the *Src64B* + *Ras^{ACT}* + *Dp110^{DN}* clonal tissue (Gi).
